# Supplementary material for: Antibody-mediated neutralization of galectin-3 as a strategy for the treatment of systemic sclerosis
Source: Nat Commun. 2023 Aug 31;14:5291. doi: 10.1038/s41467-023-41117-9 (PMC10471779; doi:10.1038/s41467-023-41117-9)
Supplement: Supplementary file 1 — Supplementary Information [file 41467_2023_41117_MOESM1_ESM.pdf]

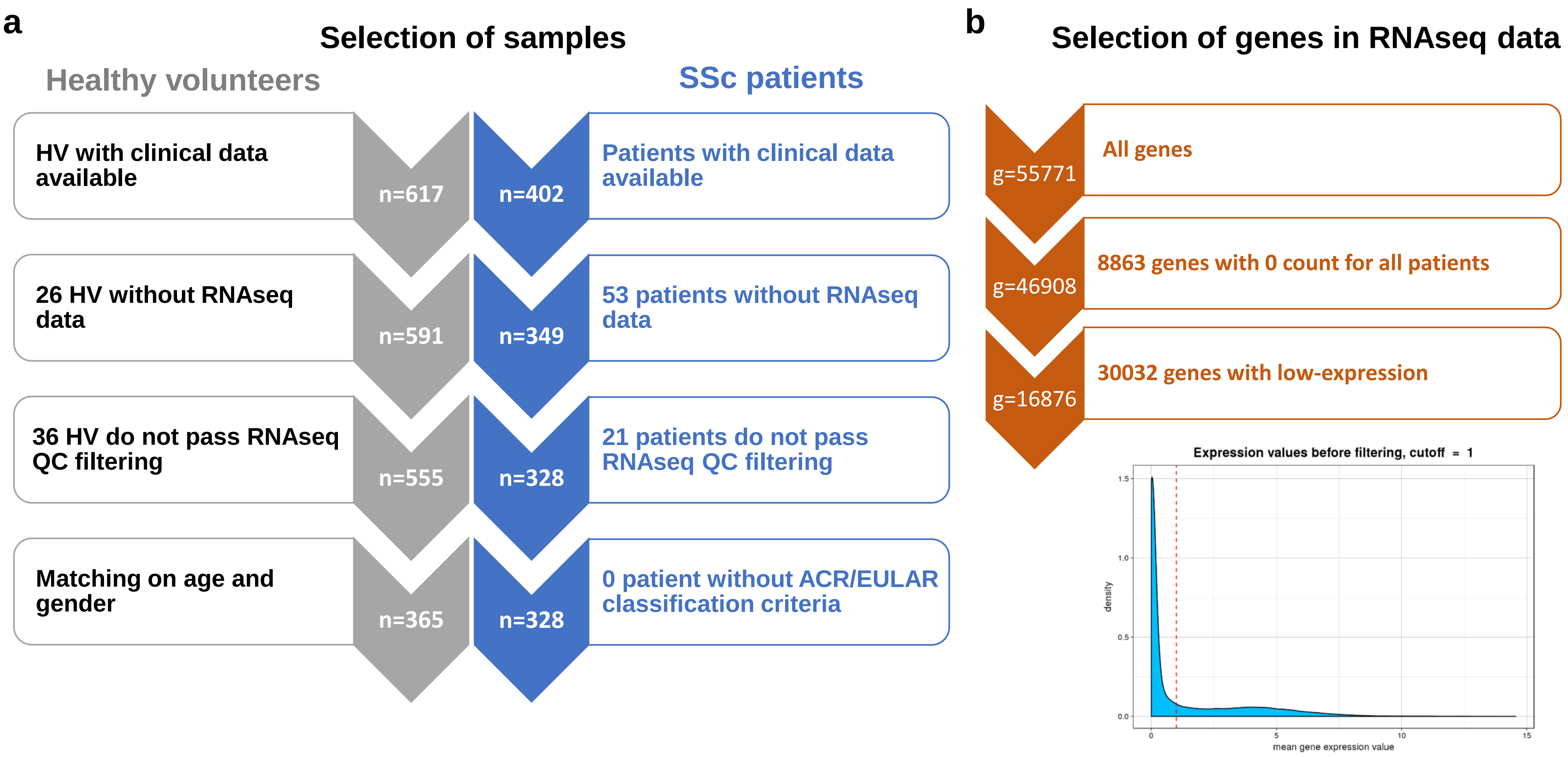

Supplementary Figure 1. RNAseq analysis flow chart.

a Flow chart summarizing the process of selection of systemic sclerosis (SSc) patients and healthy volunteers (HV) in the study. Patients and HV were selected based on the availability of clinical data. Within these patients, those without RNAseq data and who did not pass RNAseq quality control (QC) filtering were excluded.
b Flowchart summarizing the selection of genes used in the whole blood cells RNAseq data. Genes with no counts or low expression levels were excluded.

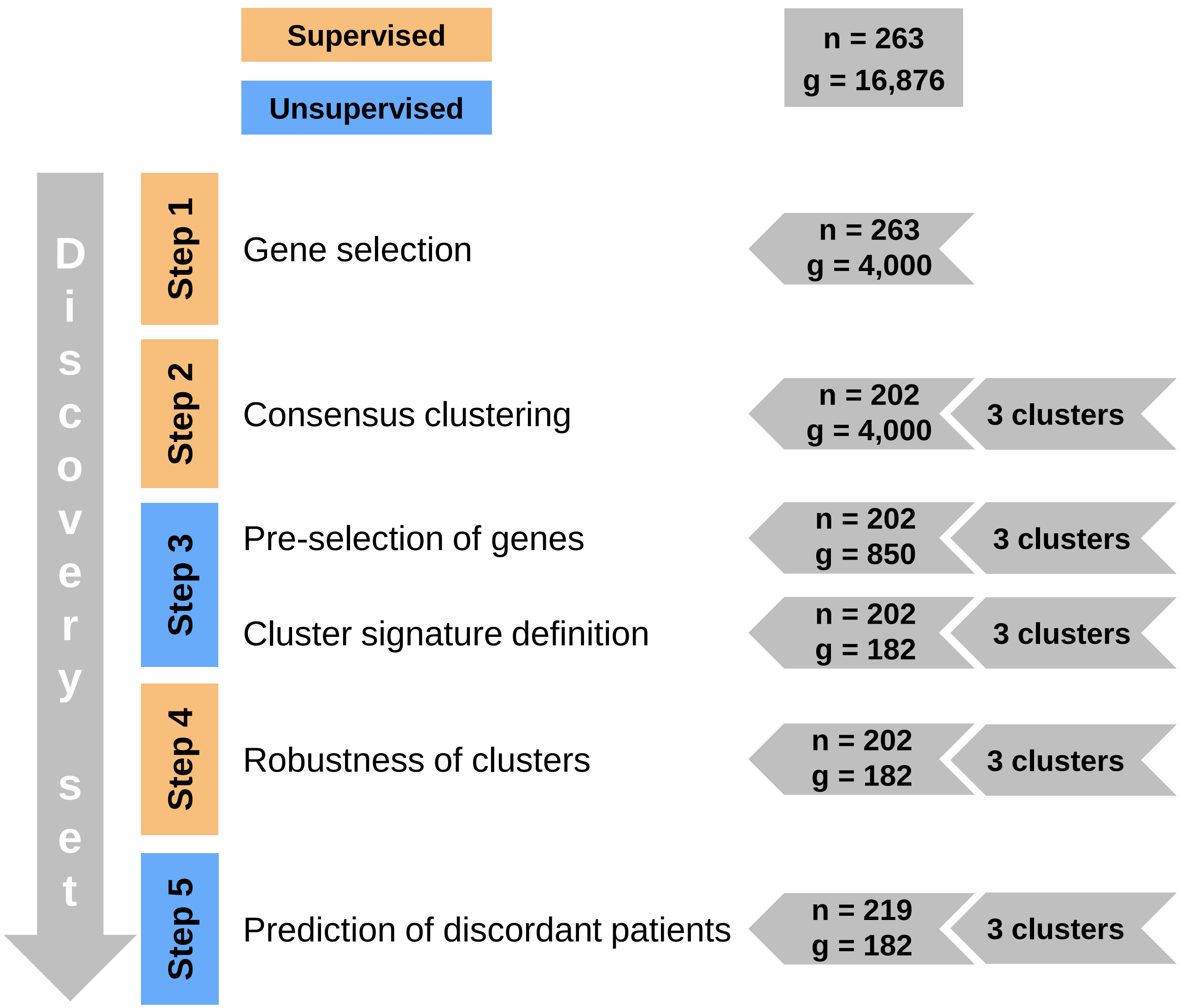

Supplementary Figure 2. Flow-chart summarizing the stepwise approach used for cluster signature definition and patient prediction in clusters.
SSc patients were classified with 3 unsupervised methods in 3 clusters. Out of the 263 patients, 202 yielded a consensus subgroup assignment in a cluster. Supervised classification identified a minimal list of 182 discriminative genes. A centroid based predictor was used to predict discordant patients.

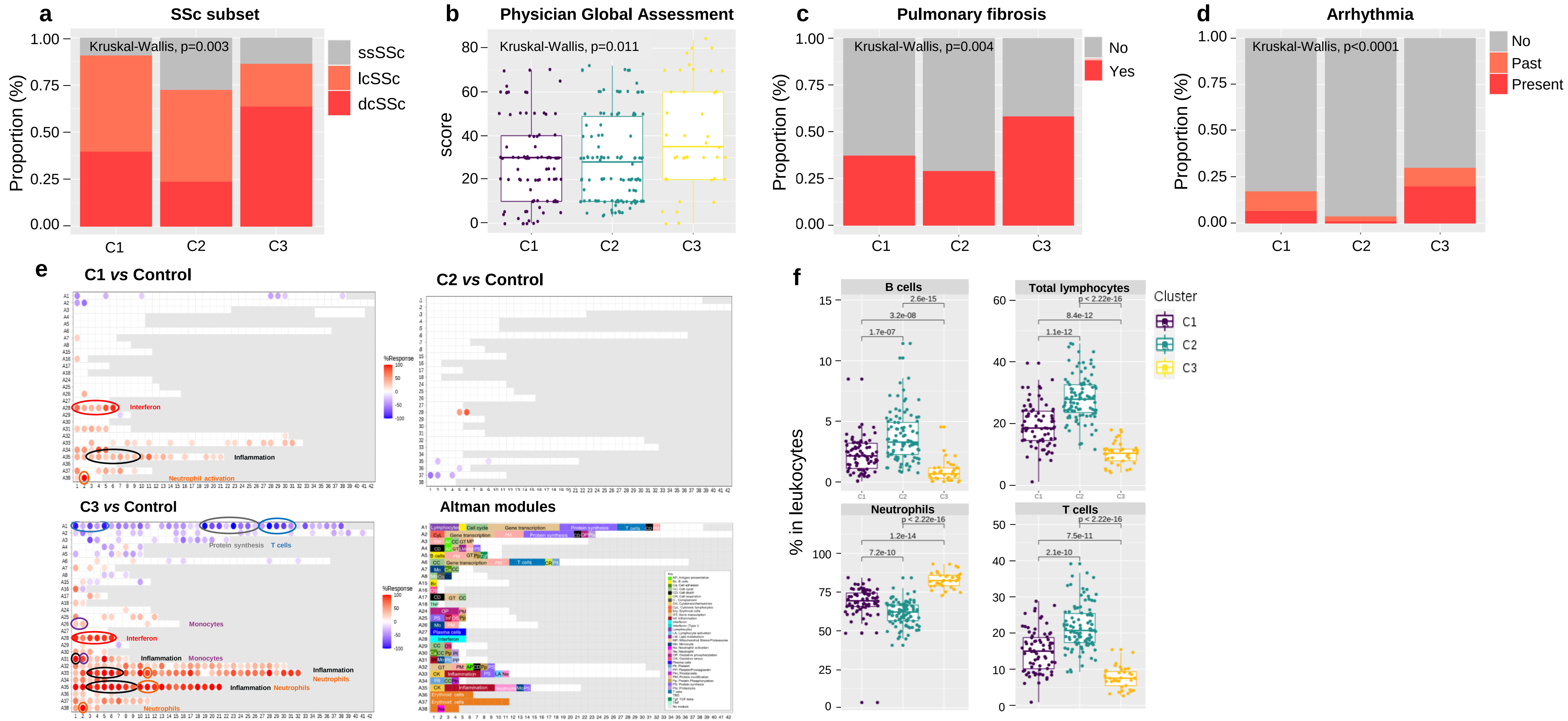

Supplementary Figure 3. Characteristics of SSc clusters. a Cluster analysis of systemic sclerosis (SSc) subset proportions collected for 141 patients (C1:43, C2:76, C3:22). **b** Cluster analysis of physician global assessment scores collected for 239 patients (C1, purple:84, C2, turquoise:114, C3, yellow:41). **c** Cluster analysis of the proportion of pulmonary fibrosis collected for 231 patients (C1:78, C2:110, C3:43). **d** Cluster analysis of the proportion of arrhythmia collected for 223 patients (C1:76, C2:107, C3:40). **e** Pathway analysis based on transcriptomic data in each cluster. The relative involvement of pathways decoded by the Altman module is reported for each SSc cluster. Intensities are displayed as colours ranging from blue for a lower response to red for a higher response as indicated in the key. **f** Cluster analysis of immune cell counts measured by flow cytometry and expressed as the percentage of total leukocytes in whole blood cells, collected for 211 patients (C1, purple:76, C2, turquoise:98, C3, yellow:37). Results in **b** and **f** are depicted as boxes and whiskers plots defined by minimal, maximal and median values in each data set. The upper whisker extends from the hinge to the largest value no further than 1.5 * IQR from the hinge (where IQR is the inter-quartile range, or distance between the first and third quartiles). The lower whisker extends from the hinge to the smallest value at most 1.5 * IQR of the hinge. Statistical analyses were performed using Kruskal-Wallis test in **b-d**, and two-tailed pairwise Wilcoxon-rank sum test in **f.**

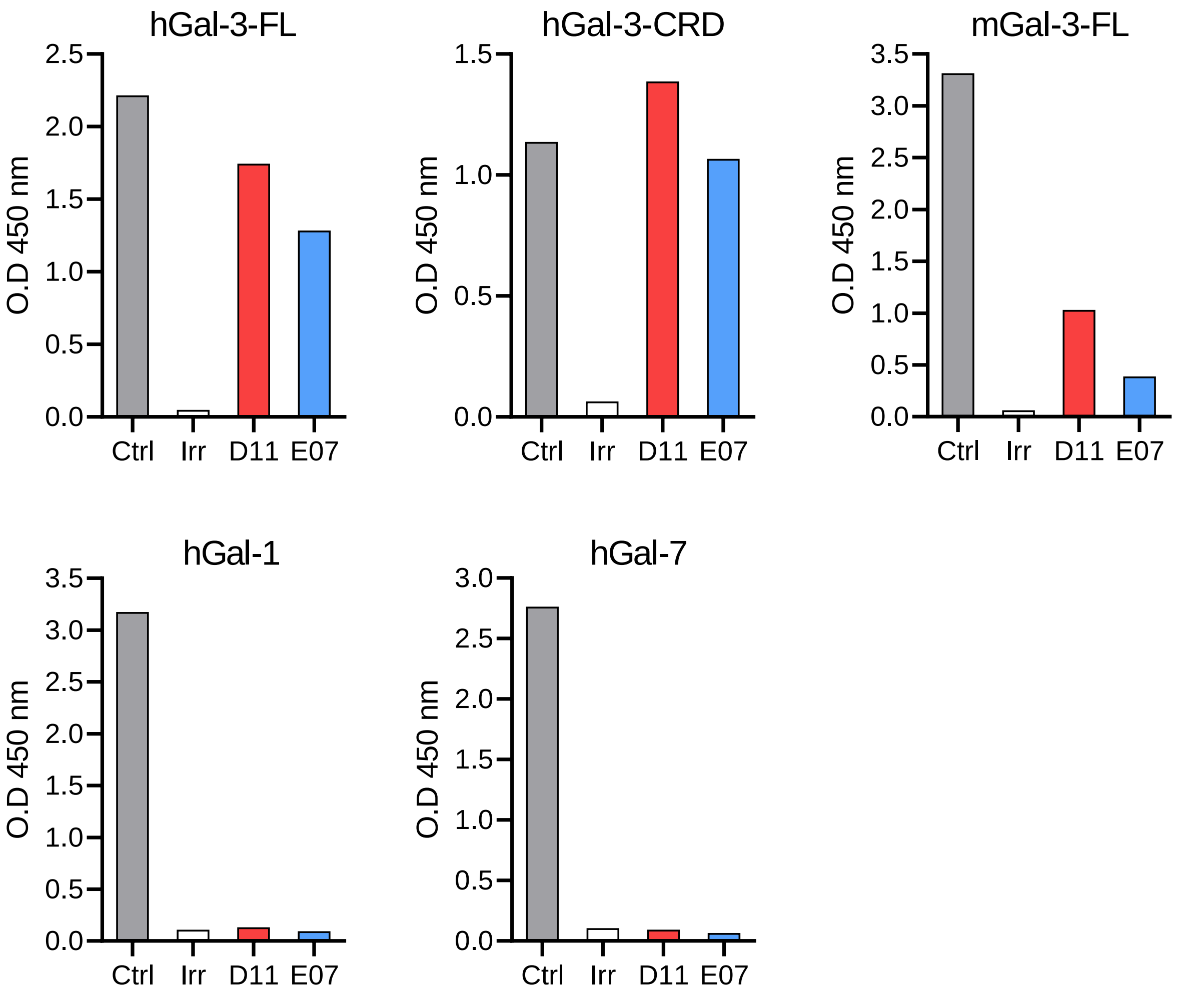

Supplementary Figure 4. Binding characteristics of lead ScFv (parent to D11 and E07 mAbs) to human (h) and mouse (m) Gal-3 constructs and their selectivity towards human Gal-1 and Gal-7 measured by ELISA. Periplasmic extracts were tested at a 1:2 dilution (D11, red; E07, blue) and the appropriate positive control (commercial antibody against Gal-3, Gal-1 or Gal-7; Ctrl, grey bars) was used at 5 µg/mL. An irrelevant ScFv (Irr) was used as negative control for each assay (white bars). Data are representative of one experiment performed during the anti Gal-3 IgG selection flow scheme and represent a single measurement per condition.

a

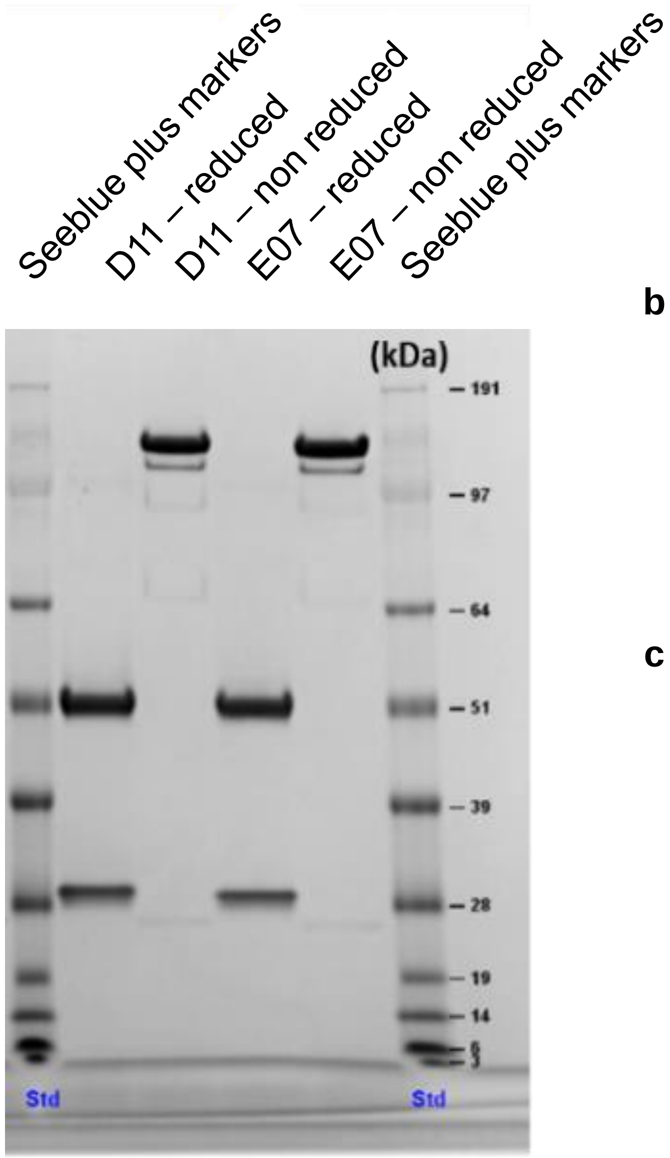

b

mAb	Theoretical mass (Da)	Measured mass (LC-MS) (Da)	Mass accuracy (ppm)	Homogeneity (SEC-MALS) (%)
**D11**	144163.82	144161.86	14	> 99
**E07**	144402.06	144400.14	13	> 99

c

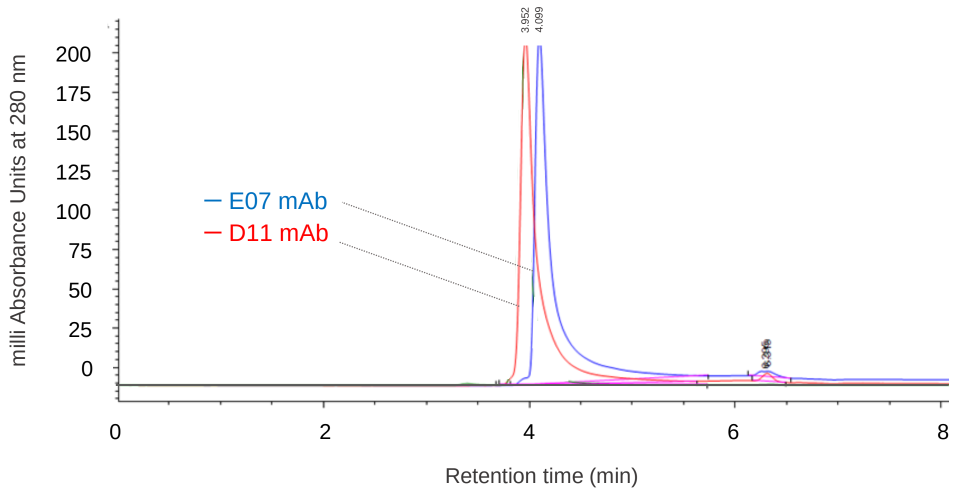

Supplementary Figure 5. Analytical characterization of D11 and E07 Gal-3 neutralizing mAbs. **a** SDS-PAGE profile; **b** Molecular mass and homogeneity values of D11 and E07 mAbs determined by liquid chromatography-mass spectrometry (LC-MS) and size exclusion chromatography with multi angle light scattering detection (SEC-MALS). **c** SEC-UV analysis of D11 mAb (red curve) and E07 mAb (blue curve). Data are representative of one experiment performed as quality control of D11 and E07 mAbs used for *in vitro* and *in vivo* experiments.

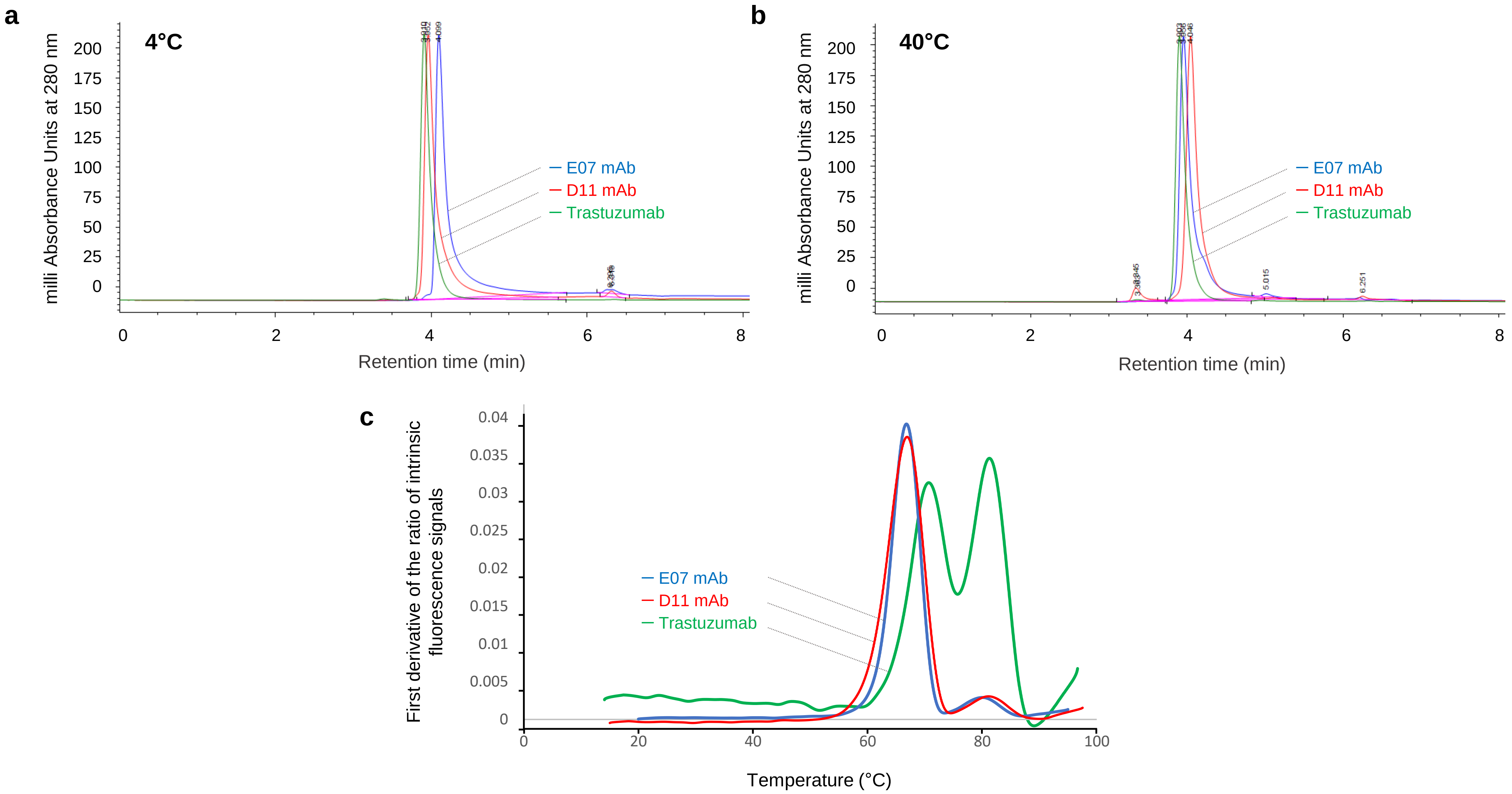

Supplementary Figure 6. Effect of temperature on D11 and E07 mAb stabilities in comparison with trastuzumab. **a** Size exclusion chromatography-UV (SEC-UV) analysis of D11 mAb (red curve), E07 mAb (blue curve) and trastuzumab (green curve) after storage at 4°C for 2 weeks. **b** SEC-UV analysis of D11 mAb, E07 mAb and trastuzumab after storage at 40°C for 2 weeks. **c** Thermal stability of D11 mAb, E07 mAb and trastuzumab assessed by nano differential scanning fluorimetry. Data are representative of one experiment performed for each separate condition.

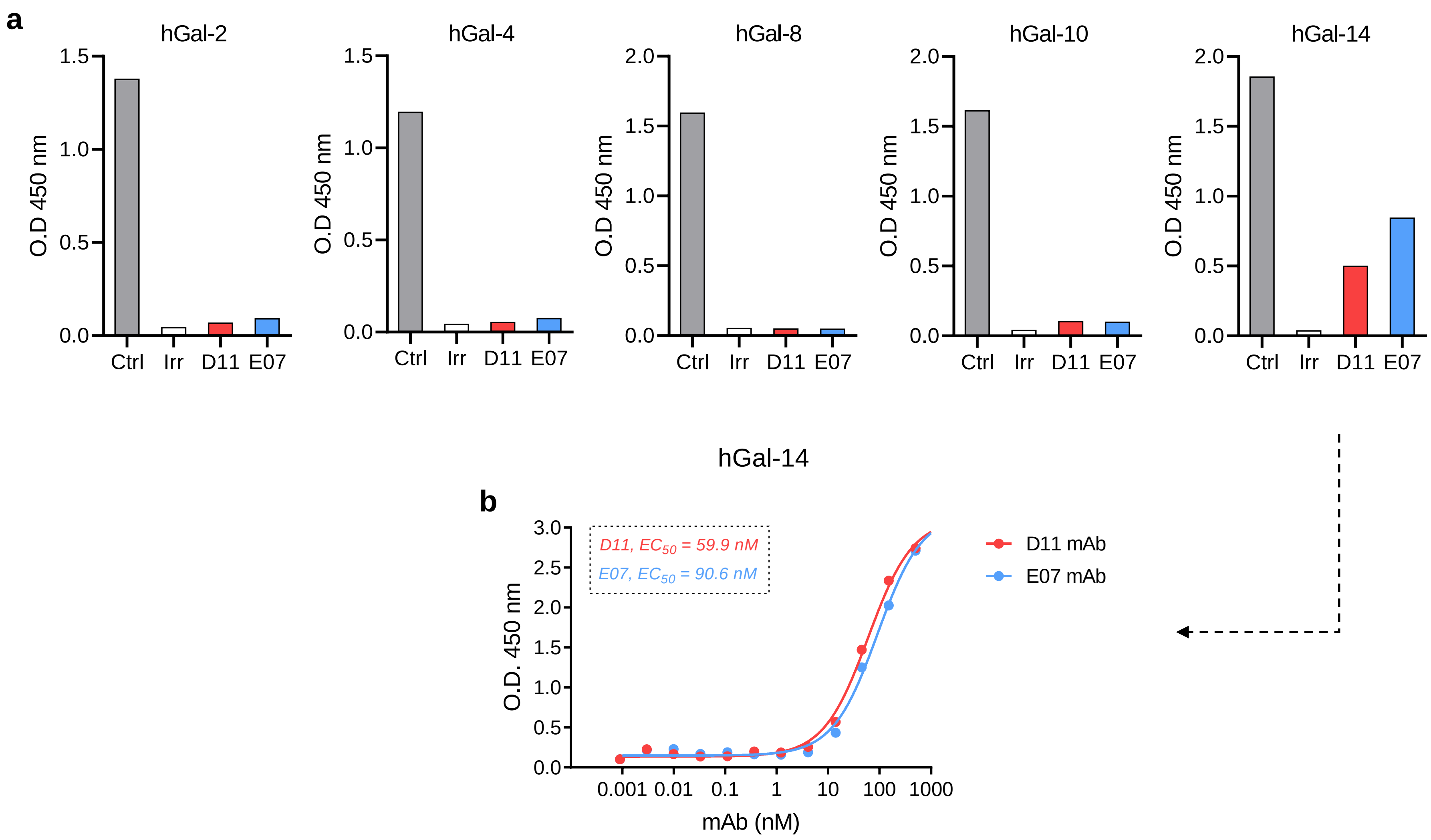

Supplementary Figure 7. Selectivity of D11 and E07 mAbs towards human Gal-2, -4, -8, -10 and -14 measured by ELISA. **a** Each mAb (D11, red; E07, blue) was tested at 10 µg/mL, and the appropriate positive control (commercial antibody against Gal-2, Gal-4, Gal-8, Gal-10 or Gal-14; Control, grey bars) was used at 5 µg/mL. An irrelevant IgG (Irr) was used as negative control for each assay. **b** Dose-response ELISA of D11 and E07 mAbs binding to hGal-14. All data are representative of one experiment and represent a single measurement per condition.

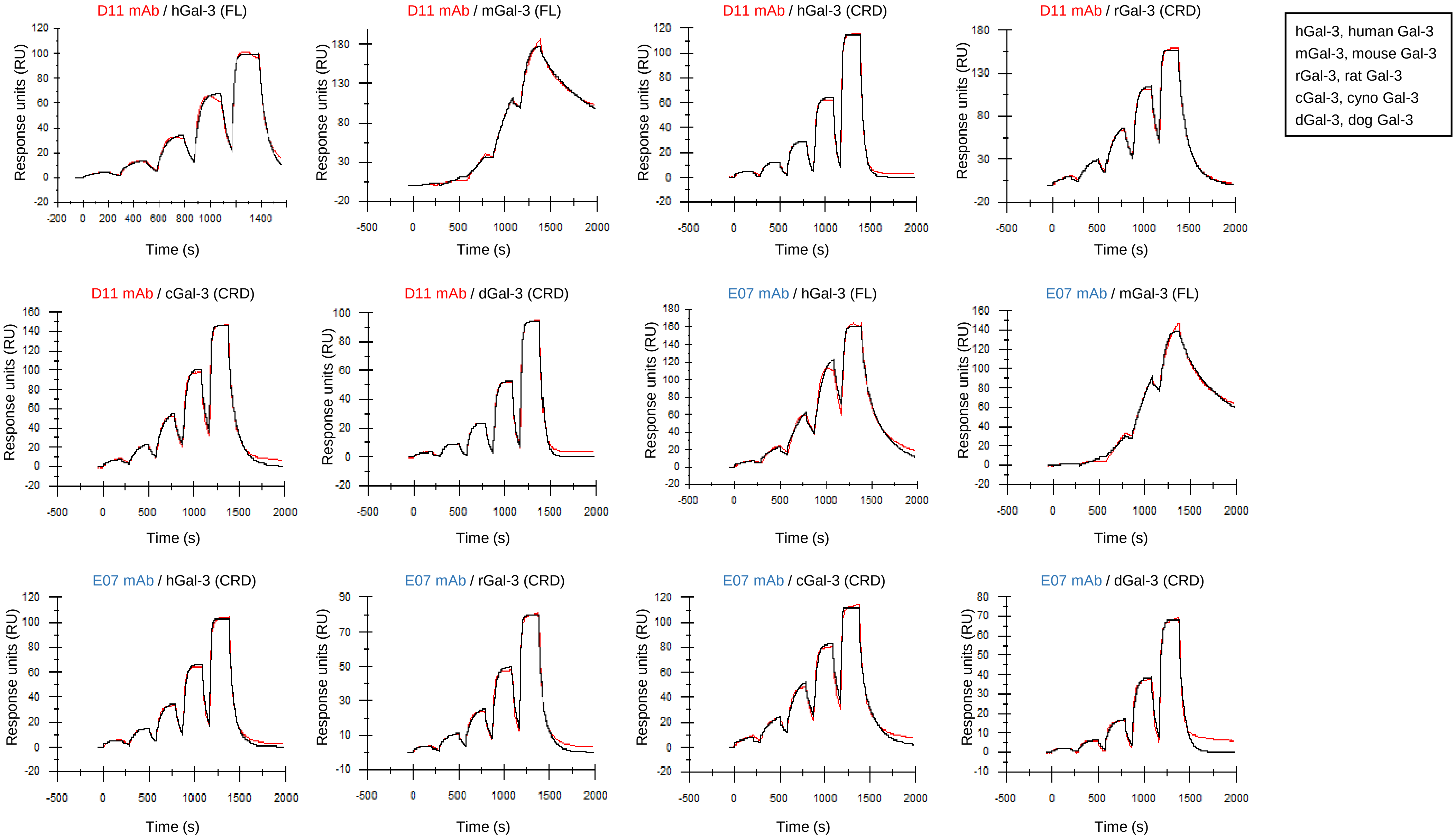

Supplementary Figure 8. SPR sensorgram binding profiles of D11 and E07 Gal-3-neutralizing mAbs to Gal-3 from multiple species. Sensorgrams depicted in this figure correspond to the experimental conditions having generated K_D values presented in Figure 4d. h, human; m, mouse; r, rat; c, cynomolgus; d, dog. Results are shown for one single experiment for each condition, using full-length (FL) Gal-3 or the carbohydrate recognition domain (CRD) of Gal-3.

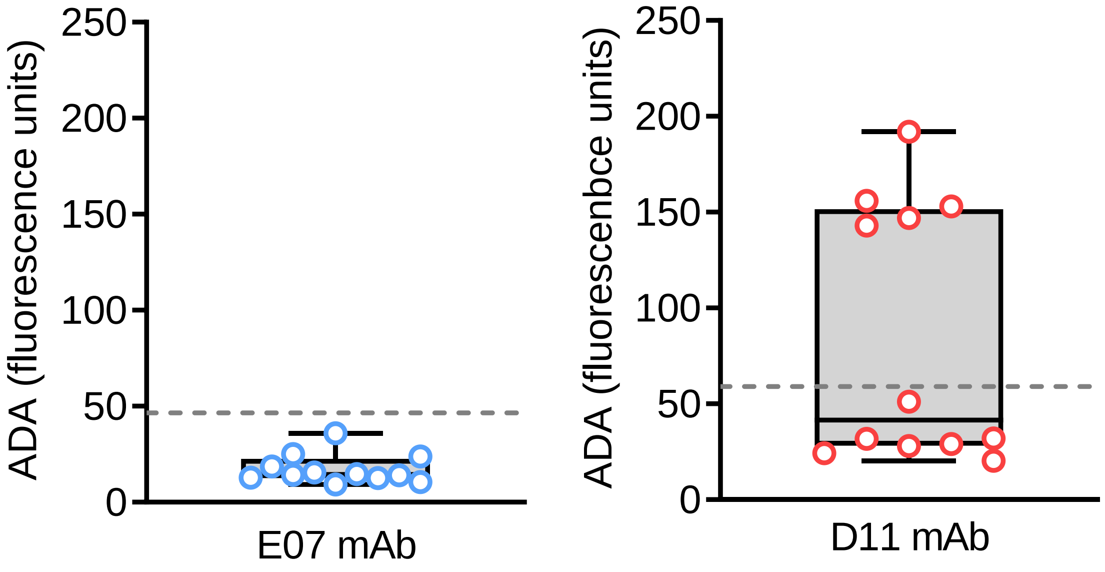

Supplementary Figure 9. Relative plasma levels of anti-drug antibodies (ADA) against E07 and D11 mAbs in the mouse model of HOCl-induced SSc at termination day. All mice treated with E07 mAb (blue open circles) or D11 mAb (red open circles) as detailed in the Methods section. Data are representative of one experiment conducted with n=12 biologically-independent mice per group. Results are depicted as boxes and whiskers plots defined by minimal, maximal and median values in each dataset. The dotted lines depict the cut-off threshold allowing to determine ADA positivity (below the line, ADA-negative; above the line, ADA-positive).

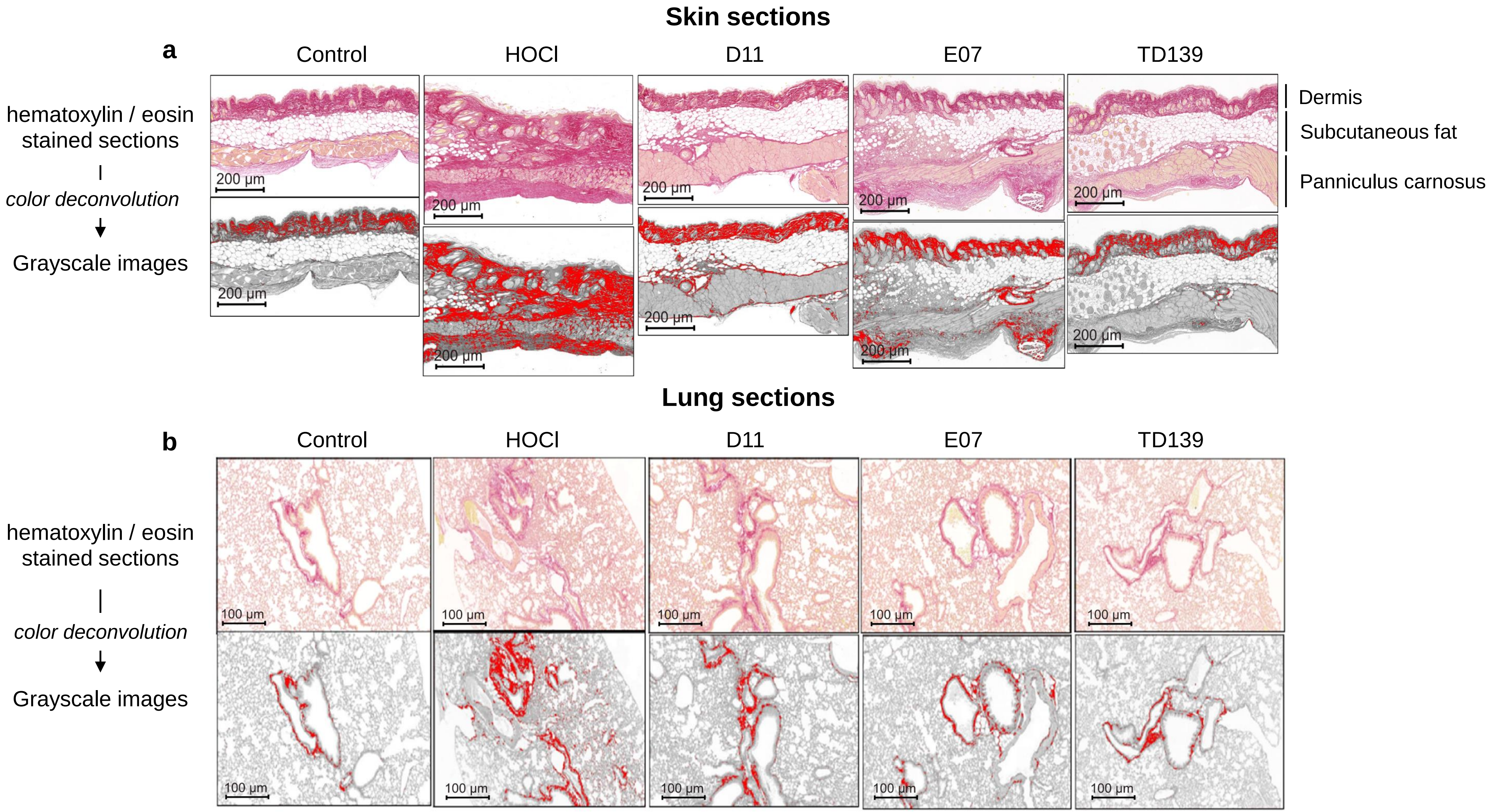

Supplementary Figure 10. Representative microphotographs of skin and lung sections after anti Gal-3 treatments in the mouse model of HOCl-induced SSc. **a** Skin sections. **b** lung sections. Upper panels for each organ correspond to hematoxylin / eosin-stained sections. Collagen deposition is revealed by picrosirius red staining and the presence of red-stained fibers in the grayscale images in the lower panels for each tissue. Images scanned at 20x magnification. Each microphotograph is representative of one skin or lung section in its representative group: three skin sections per mouse with n=12 biologically-independent mice per group, except for the control group where n=10; three lung sections per mouse with n=12 biologically-independent mice per group, except for the control, HOCl and D11 groups where n=11. Statistical analyses of skin and lung deposition in all groups are presented in Figures 5d and 5e, respectively.

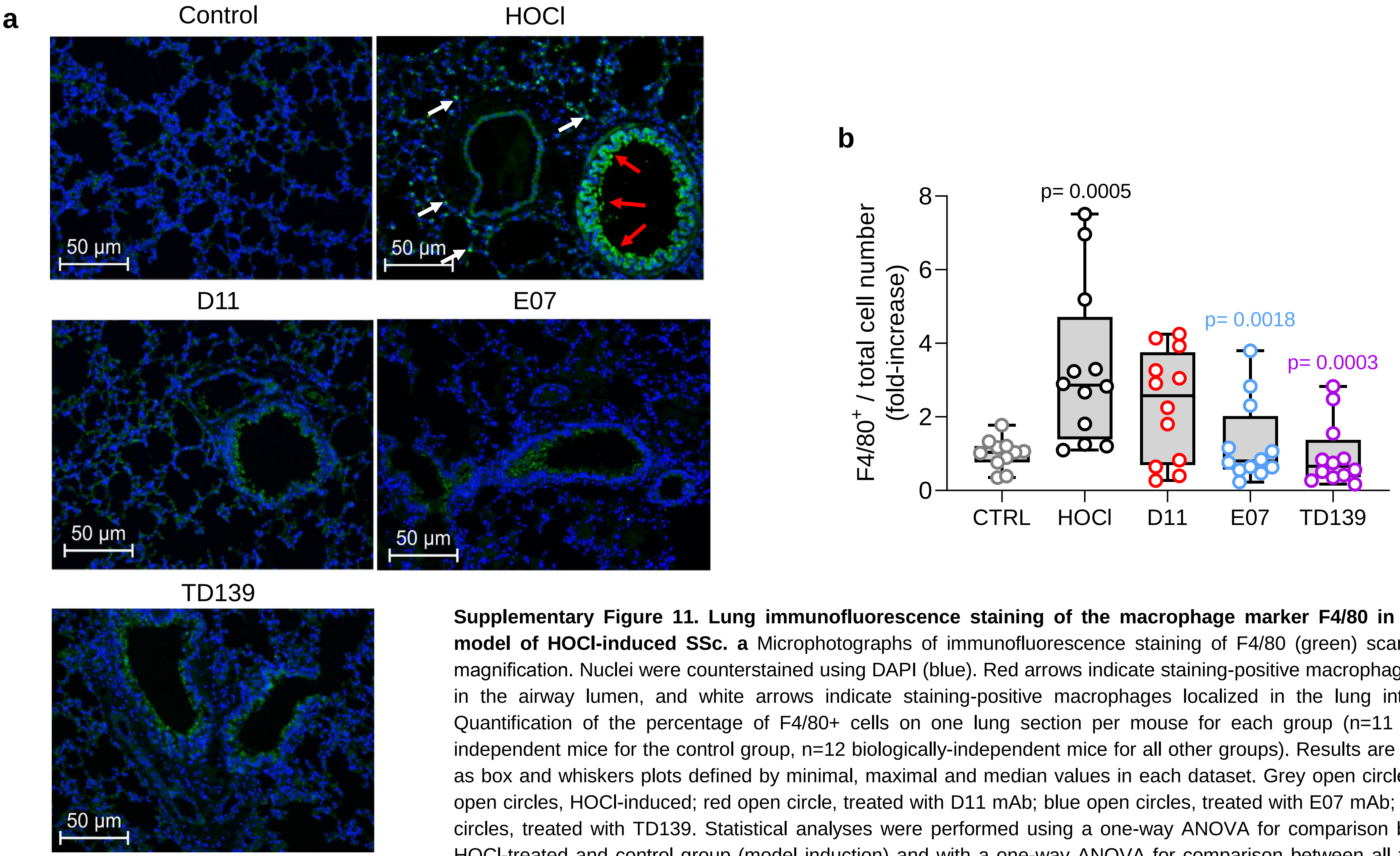

Supplementary Figure 11. Lung immunofluorescence staining of the macrophage marker F4/80 in the mouse model of HOCl-induced SSc. a Microphotographs of immunofluorescence staining of F4/80 (green) scanned at 40x magnification. Nuclei were counterstained using DAPI (blue). Red arrows indicate staining-positive macrophages localized in the airway lumen, and white arrows indicate staining-positive macrophages localized in the lung interstitium. **b** Quantification of the percentage of F4/80+ cells on one lung section per mouse for each group (n=11 biologically-independent mice for the control group, n=12 biologically-independent mice for all other groups). Results are represented as box and whiskers plots defined by minimal, maximal and median values in each dataset. Grey open circles, ctrl; dark open circles, HOCl-induced; red open circle, treated with D11 mAb; blue open circles, treated with E07 mAb; purple open circles, treated with TD139. Statistical analyses were performed using a one-way ANOVA for comparison between the HOCl-treated and control group (model induction) and with a one-way ANOVA for comparison between all tested items and their HOCl control group, using Dunnett's adjustment.

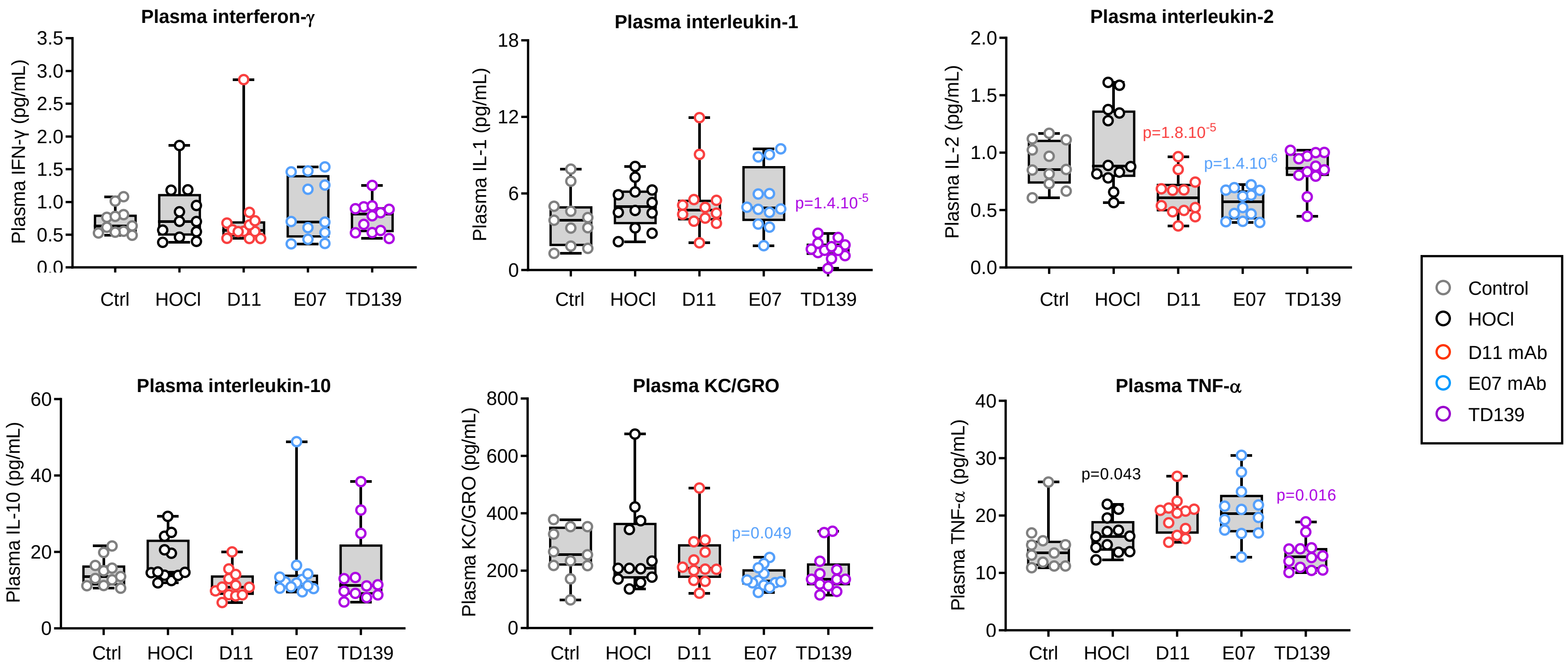

Supplementary Figure 12. Effect of anti Gal-3 treatments on individual plasma cytokine levels in the mouse model of HOCl-induced SSc. Control (Ctrl, light grey open circles) and HOCl (dark open circles) groups represent vehicle-receiving and pathology-induced mice, respectively. D11 (red open circles), E07 (blue open circles) and TD139 purple open circles) groups represent anti Gal-3 treatments administered in HOCl-induced mice as described in the Methods section. Data are representative of one experiment conducted with 11 biologically-independent mice for the control group and n=12 biologically-independent mice for all other groups, except in specific cases due to experimental sample loss or unavailability, as detailed in the source data file. Statistical analyses were performed on log-transformed data using a mixed ANCOVA model for comparison between the HOCl and control group, and using a mixed ANCOVA model for comparison between all tested items and their HOCl control group, using Dunnett's adjustment.

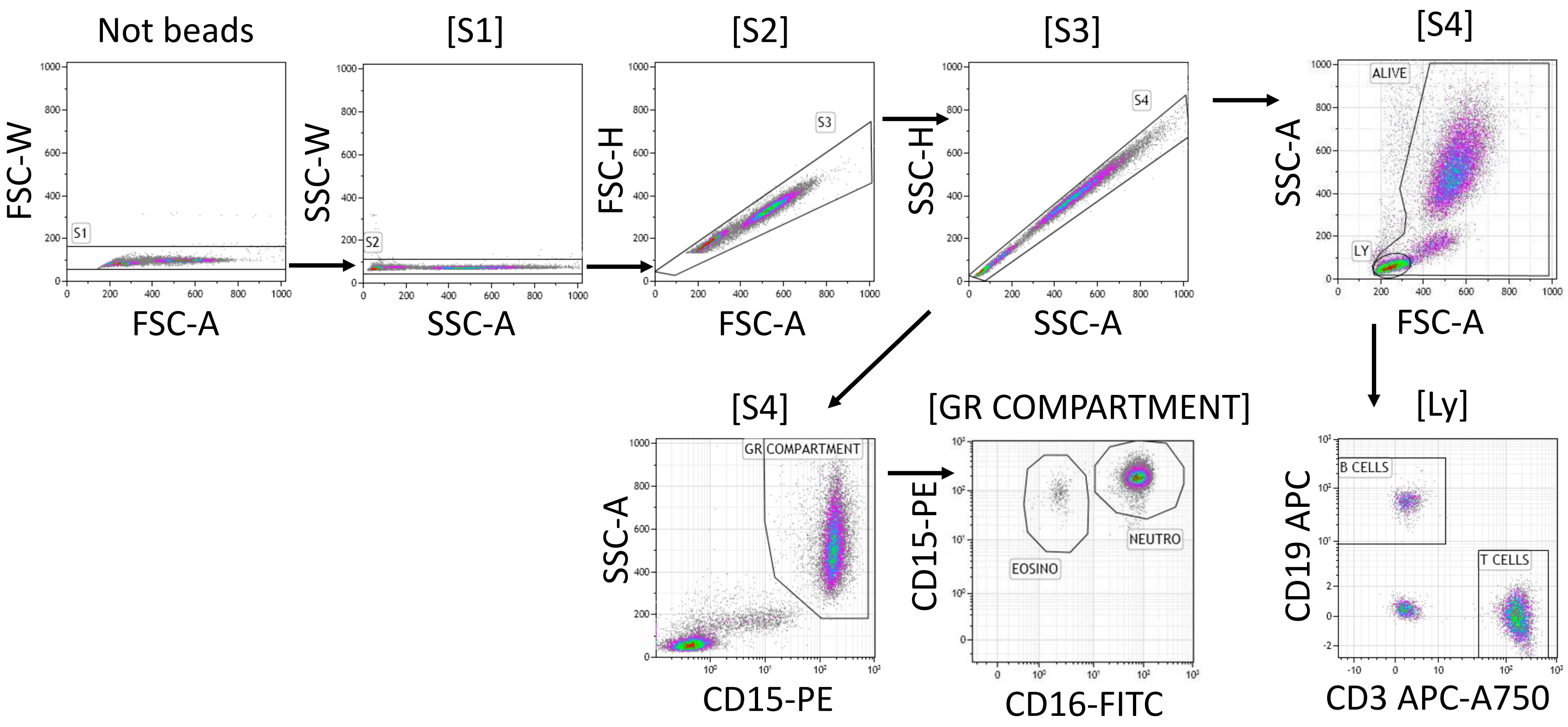

Supplementary Fig. 13. Gating strategy for immune cell populations in the human PRECISESADS cohort. A representative fluorescence activated cell sorting (FACS) plot from multi centre flow cytometric analyses is shown (adapted from reference 61). Gating panels are representative of the gating strategy used to produce the results presented in Figure 2d and Supplementary Figure 3f. $CD15^{hi}CD16^{hi}$ = neutrophils; FSC-A^{low} SSC-A^{low} = total lymphocytes, $CD19^{+}$ = B cells and $CD3^{+}$ = T cells; $CD19^{+}$ B cells and $CD3^{+}$ T cells gated on single cells (S1 to S3) and live cells (S4). Fluorochromes: PE, phycoerythrin; FITC, Fluorescein isothiocyanate; APC, allophycocyanin; APC-A750, APC-alexa fluor 750.

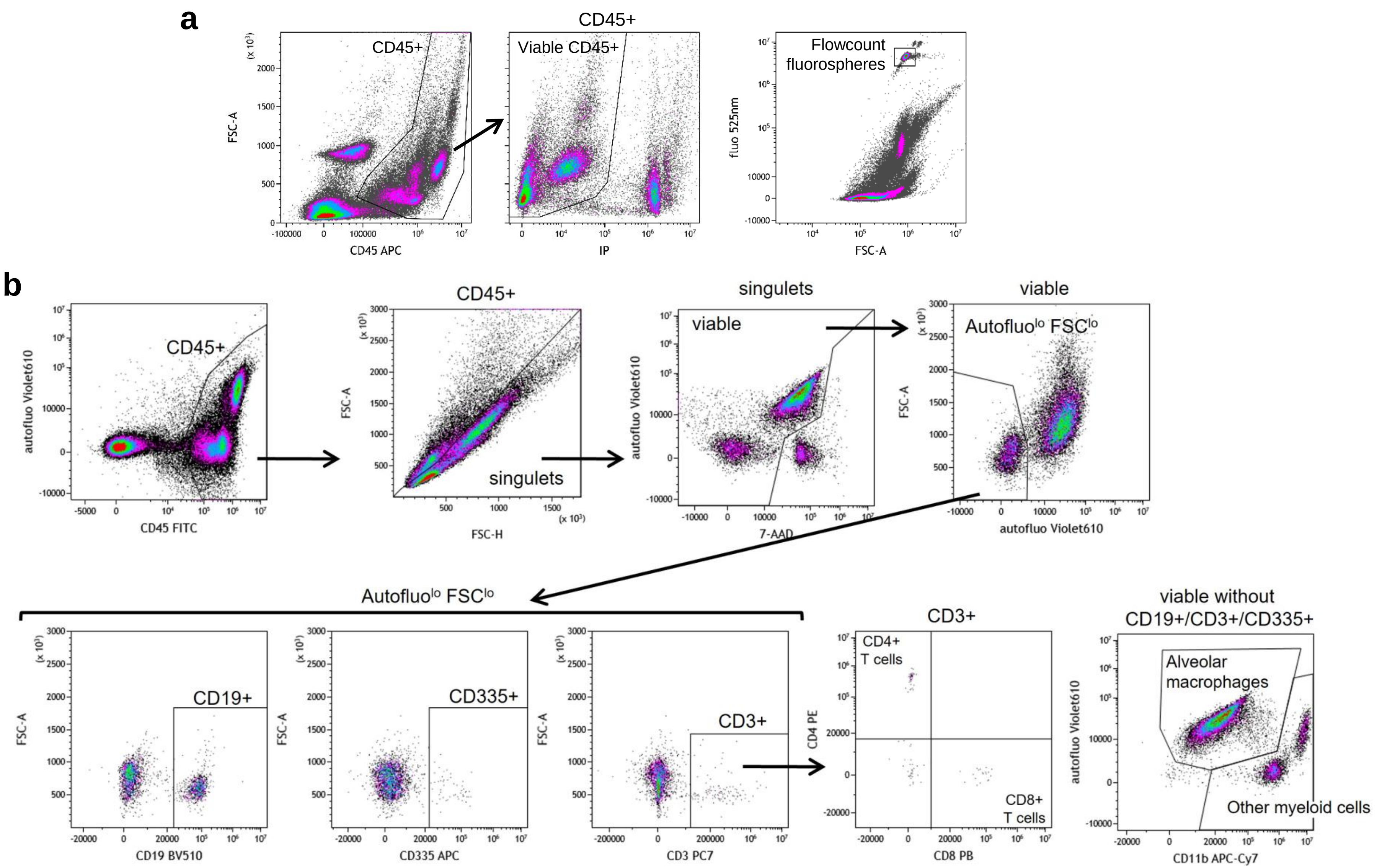

Supplementary Figure 14. Gating strategy for BAL immunophenotyping in the mouse model of HOCl-induced SSc. a Gating strategy for the numeration of viable CD45+ cells in BAL fluid. **b** Gating strategy for phenotypic analysis of the cellular content in BAL fluid. Gating panels **a** and **b** are from a mouse of the control group and are representative examples of the gating strategy used to produce the results presented in Figure 5h. CD45+ cells = immune cells; CD19+ cells = B cells; CD335+ cells = NK cells; CD3+ CD4+ cells = CD4+ T cells; CD3+ CD8+ cells = CD8+ T cells. Among CD19- CD3- CD335- cells: alveolar macrophages = autofluorescence^{high} CD11b$^{-/low}$ and other myeloid cells (monocytes, dendritic cells, neutrophils and eosinophils) = autofluorescence$^{low/int}$ CD11b$^{+/high}$. Fluorochromes: APC, allophycocyanin; FITC, Fluorescein isothiocyanate; BV510, Brilliant Violet 510; PC7, phycoerythrin-cyanine7; PB, Pacific Blue; PE, Phycoerythrin; APC-Cy7, allophycocyanin-Cyanin7.
