# Supplementary material for: Antibody-mediated neutralization of galectin-3 as a strategy for the treatment of systemic sclerosis
Source: Nat Commun. 2023 Aug 31;14:5291. doi: 10.1038/s41467-023-41117-9 (PMC10471779; doi:10.1038/s41467-023-41117-9)
Supplement: Supplementary file 3 — Description of additional supplementary files [file 41467_2023_41117_MOESM3_ESM.pdf]

Description of Additional Supplementary Files Document

Supplementary Data 1. List of Gal-3 interactants.

A non-redundant list of Gal-3 interacting proteins was compiled by bioinformatics by querying the GPS-prot database, allowing the referencing of 210 Gal-3 interactants, and the Ingenuity Pathway Analysis database, allowing the referencing of 276 Gal-3 interactants. Merging the 2 databases finally led to the identification of 307 proteins, listed by alphabetical order in column A. Their location determined from the Ingenuity Pathway Analysis (IPA) database is provided in column B. Among this list, 59 Gal-3 interactants were found absent in datasets of the PRECISESADS SSc cohort (column B). An alternative classification of the 307 Gal-3 interactants by location rather than alphabetical order is provided in columns D and E.

Supplementary Data 2. Differential gene expression of Gal-3 interactants in clusters of the PRECISESADS SSc cohort.

Differential gene expression between each cluster and healthy volunteers is reported for genes for which statistical significance was reached. Cluster 3 *versus* healthy volunteers, yellow; Cluster 1 *versus* healthy volunteers, blue; Cluster 2 *versus* healthy volunteers, purple. Statistical analyses were performed using a linear model (lmFit function from limma v3.46.0 R package) on vst transformation gene expression dataset. The resulting p-values were adjusted for multiple hypothesis testing and filtered to retain differentially expressed genes with false discovery rate (FDR) adjusted p-values ≤ 0.05 and an absolute fold-change (|FC|) ≥ 1.3.

Supplementary Data 3. Differential gene expression analysis in the mouse model of HOCl-induced systemic sclerosis.

Differential gene expression between groups is reported for genes for which statistical significance was reached. Pathological HOCL-induced mice *versus* control untreated mice, columns A-C; D11 mAb-treated mice *versus* pathological HOCL-induced mice, columns D-F; E07 mAb-treated mice *versus* pathological HOCL-induced mice, columns G-I; TD139-treated mice *versus* pathological HOCL-induced mice, columns J-L; Statistical analyses were performed using a linear model (lmFit function from limma v3.46.0 R package) on vst transformation gene expression dataset. The resulting p-values were adjusted for multiple hypothesis testing and filtered to retain differentially expressed genes with false discovery rate (FDR) adjusted p-values ≤ 0.05 and an absolute fold-change (|FC|) ≥ 1.3.

Supplementary Data 4. List of pathological genes modulated in HOCl-induced mice versus control mice and reversed after E07 mAb treatment.

Differential gene expression is reported for genes for which statistical significance was reached. Statistical analyses were performed using a linear model (lmFit function from limma v3.46.0 R package) on vst transformation gene expression dataset. The resulting p-values were adjusted for multiple hypothesis testing and filtered to retain differentially expressed genes with false discovery rate (FDR) adjusted p-values ≤ 0.05 and an absolute fold-change (|FC|) ≥ 1.3.
