# Supplementary material for: Antibody-mediated neutralization of galectin-3 as a strategy for the treatment of systemic sclerosis
Source: Nat Commun. 2023 Aug 31;14:5291. doi: 10.1038/s41467-023-41117-9 (PMC10471779; doi:10.1038/s41467-023-41117-9)
Supplement: Supplementary file 8 — Reporting Summary [file 41467_2023_41117_MOESM8_ESM.pdf]

nature portfolio

Corresponding author(s): De Ceuninck F

Last updated by author(s): Aug 3, 2023

Reporting Summary

Nature Portfolio wishes to improve the reproducibility of the work that we publish. This form provides structure for consistency and transparency in reporting. For further information on Nature Portfolio policies, see our Editorial Policies and the Editorial Policy Checklist.

Statistics

For all statistical analyses, confirm that the following items are present in the figure legend, table legend, main text, or Methods section.

n/a	Confirmed	
☐	☒	The exact sample size (*n*) for each experimental group/condition, given as a discrete number and unit of measurement
☐	☒	A statement on whether measurements were taken from distinct samples or whether the same sample was measured repeatedly
☐	☒	The statistical test(s) used AND whether they are one- or two-sided<br>*Only common tests should be described solely by name; describe more complex techniques in the Methods section.*
☐	☒	A description of all covariates tested
☐	☒	A description of any assumptions or corrections, such as tests of normality and adjustment for multiple comparisons
☐	☒	A full description of the statistical parameters including central tendency (e.g. means) or other basic estimates (e.g. regression coefficient) AND variation (e.g. standard deviation) or associated estimates of uncertainty (e.g. confidence intervals)
☐	☒	For null hypothesis testing, the test statistic (e.g. *F*, *t*, *r*) with confidence intervals, effect sizes, degrees of freedom and *P* value noted<br>*Give P values as exact values whenever suitable.*
☒	☐	For Bayesian analysis, information on the choice of priors and Markov chain Monte Carlo settings
☐	☒	For hierarchical and complex designs, identification of the appropriate level for tests and full reporting of outcomes
☒	☐	Estimates of effect sizes (e.g. Cohen's *d*, Pearson's *r*), indicating how they were calculated

Our web collection on statistics for biologists contains articles on many of the points above.

Software and code

Policy information about availability of computer code

Data collection	No software used for data collection
Data analysis	- Human study:<br>Except when indicated, data analyses were carried out using either an assortment of R system software (http://www.R-project.org, V4.0.1) packages including those of Bioconductor v3.17 or original R code. R packages are indicated when appropriate.<br>In order to analyze RNAseq data, first, bcl2fastq2 Conversion Software v2.20 was used to demultiplex sequencing data and convert BCL files. Quality control was obtained with FastQC tools v0.11.18 and adapters were removed with Cutadapt v1.18.<br>Transcriptome alignment was done with STAR v2.5.2b on GENCODE v19 annotation (hg19) and read counts were obtained with RSEM v1.2.31. For normalizations and batch correction, read counts were normalized by the variance stabilizing transformation vst function from DESeq2 v1.30.0 R package. To reduce the effect of the RIN, a correction was applied using the ComBat function from sva v3.38.0 R package, after categorization of RIN values into 7 classes: [6.5-7.0], [7.0-7.5], [7.5-8.0], [8.0-8.5], [8.5-9.0], [9.0-9.5], [9.5-10].<br>To determine the number of clusters of patients, a consensus clustering between three methods was performed: (i) Agglomerative Hierarchical Clustering (hclust function from stats v4.0.2 R package) with Pearson correlation as a similarity measure and the Ward's linkage method, (ii) K-means clustering (k means function from stats R package) with 4 groups and (iii) Gaussian mixture clustering (mclust function from mclust v5.4.6 R package).<br>The top discriminating genes were defined with randomForest function from randomForest v4.6-14 R package.<br>Heatmaps were obtained with ComplexHeatmap v2.6.2 R package.<br>To identify genes differentially expressed between SSc subgroups and HV we performed a linear model (ImFit function from limma v3.46.0 R package) on vst transformation gene expression dataset. Resulting p-values were adjusted for multiple hypothesis testing and filtered to retain differentially-expressed genes with a False Discovery Rate (FDR) adjusted p-value 0.05 and a I Fold-Change (FC) I 1.3.<br>Enrichment analysis was performed with BloodGen3Module v0.99.36 R package.

Canonical pathway analysis was performed with Ingenuity Pathway Analysis (IPA, Release Date: 2020-06-01).
The code for the clustering of patients has been deposited to github (https://github.com/psBiostat/GAL3_PAPER.git) and linked to Zenodo (https://doi.org/10.5281/zenodo.8205835).
- Mouse HOCI study:
All statistical analyses, except for RNAseq studies, were performed using SAS Version 9.4.
Flow cytometry data were analyzed using Kaluza software v2.1.
Lung and skin histogy slides were analyzed using the web-based ImageJ software v1.53t. The number of F4/80-expressing cells was quantified using the QuPath software (v.0.4.1).
mAb concentrations in PK analyses were calculated using the Gyrolab software (no version number).
Results from RNAseq studies were analyzed a linear model (lmFit function from limma v3.46.0 R package) on vst transformation gene expression dataset. The resulting p-values were adjusted for multiple hypothesis testing and filtered to retain differentially-expressed genes with a FDR adjusted p-value ≤ 0.05 and a |FC| value ≥ 1.3. Quality control was obtained with FastQC tools v0.11.18. Read counts were normalized by the variance stabilizing transformation vst function from the DESeq2 R package v1.30.0
Canonical pathway analysis was performed with Ingenuity Pathway Analysis (IPA, Release Date: 2020-06-01).
- mAb characterization:
mAbs homogeneities after SEC-MALS experiments were analyzed using Astra software v3.14.
Binding kinetics experiments by SPR were analyzed with the BiacoreT200 evaluation software v3.2.

For manuscripts utilizing custom algorithms or software that are central to the research but not yet described in published literature, software must be made available to editors and reviewers. We strongly encourage code deposition in a community repository (e.g. GitHub). See the Nature Portfolio guidelines for submitting code & software for further information.

Data

Policy information about availability of data

All manuscripts must include a data availability statement. This statement should provide the following information, where applicable:

- Accession codes, unique identifiers, or web links for publicly available datasets
- A description of any restrictions on data availability
- For clinical datasets or third party data, please ensure that the statement adheres to our policy

Human raw data are the property of the PRECISESADS consortium and are protected under the European General Data Protection Regulation (GDPR). The PRECISESADS Consortium committed to secure patient data access through the ELIXIR platform. This commitment was formerly given by written to all patients at the end of the project and to the involved Ethical Committees. PRECISESADS data hosted by ELIXIR Luxembourg are available under a controlled access at the permanent link: https://doi.org/10.17881/th9v-xt85. The access procedure is described on the ELIXIR data landing page. Access can be requested to the data stewardship team of ELIXIR Luxembourg via lcsb-datastewards@uni.lu. The future use of the Project database was framed according to the scope of the patient information and consent forms, where the use of patient data is open to scientific research in autoimmune diseases, not-for-profit use only. ELIXIR reviews applicants requests and prepares Data Access Committee's decisions on access to Data, communicates such decisions to the Data Providers, who have 10 days to exercise their right to veto; otherwise access is granted to the User.
Sequencing data in the mouse HOCI model are available on the GEO repositery under the accession number GSE226063. Mouse RNAseq data were processed using the mouse mm39 reference genome (https://www.ncbi.nlm.nih.gov/assembly/GCF_000001635.27/).
The source data underlying all figures and supplementary figures are provided as a Source Data file.

Research involving human participants, their data, or biological material

Policy information about studies with human participants or human data. See also policy information about sex, gender (identity/presentation), and sexual orientation and race, ethnicity and racism.

Reporting on sex and gender	Patients with systemic sclerosis (SSc) comprised 212 women and 37 men representing 85% and 15% of patients, respectively.<br>Healthy volunteers (HV) comprised 307 women and 58 men, representing 84% and 16% of HV, respectively.<br>Sex rather that gender was considered for women and men, based on self-reporting.
Reporting on race, ethnicity, or other socially relevant groupings	Participants were categorized on the basis of their race as reported in Table 1.<br>Other socially/constructed variables such as socioeconomic status were not considered.
Population characteristics	Patients with systemic sclerosis (SSc) and healthy volunteers (HV) matched on age and sex were obtained from the European multi-center cross-sectional study of the PRECISESADS IMI consortium. We analyzed 249 SSc patients and 365 HV, recruited in 19 institutions from 9 European countries and enrolled in the cross-sectional cohort of the PRECISESADS IMI project. The median age for patients with SSc was 60 years (Q1 quartile 50; Q3 quartile 68) and the median age for HV was 53 years (Q1 quartile 47; Q3 quartile 59).<br>Patients were treated with different medication such as hydroxychloroquine, immunosuppressants, biologicals, steroids or systemic antibiotics as reported in Table 1.
Recruitment	Recruitment was performed between December 2014 and October 2017 involving 19 institutions in 9 countries (Austria, Belgium, France, Germany, Hungary, Italy, Portugal, Spain and Switzerland). Patients were identified as being eligible to the study based on the 2013 classification criteria for systemic sclerosis defined by the american college of rheumatology (ACR)/ european ligue against rheumatism (EULAR) joint committee (van den Hoogen F, et al., classification criteria for systemic sclerosis: an American college of rheumatology/European league against rheumatism collaborative initiative. Ann Rheum Dis. 2013;72(11):1747-55) mentioned in the manuscript as reference 62. Healthy volunteers matched on age and sex and SSc participants were all over the age of 18 years and provided informed written consent for recruitment in the study. The PRECISESADS cohort participants did not receive any compensation.<br>No bias of selection has been identified.

Ethics oversight	The Ethical Review Boards of the participating institutions approved the protocol of the cross-sectional study. All patients and HV gave written informed consent for the study that was approved by local ethical committees. The cross-sectional study (registered as NCT02890121 in ClinicalTrials.com) adhered to the standards set by the International Conference on Harmonization and Good Clinical Practice (ICH-GCP) and to the ethical principles that have their origin in the Declaration of Helsinki (2013). The protection of the confidentiality of records that could identify the included subjects is ensured by the EU Directive 2001/20/EC and the applicable national and international requirements relating to data protection in each participating country.<br>Local ethical committees of the participating institutions:<br>Comitato Etico Milano, Italy; Comité de Protection des Personnes Ouest VI Brest, France; Louvain, Comité d'Ethique Hospitalo-Facultaire, Belgium; Comissao de ética para a Saude, CES do CHP Porto, Portugal; Comité Ética de Investigación Clínica del Hospital Clínic de Barcelona, Spain; Commissie Medische Ethiek UZ KU Leuven/Onderzoek, Belgium; Geschaftsstelle Ethikkommission, Cologne, Germany; Ethikkommission Hannover, Germany; Ethik Kommission. Borschkegasse, Vienna, Austria; Comité de Ética e la Investigación de Centro de Granada, Spain; Commission Cantonale d'éthique de la recherche Hopitaux universitaires de Genève, Switzerland; Csongrad Megyei Kormanyhivatal, Szeged, Hungary; Ethikkommission, Berlin, Germany; Andalusian Public Health System Biobank, Granada, Spain.

Note that full information on the approval of the study protocol must also be provided in the manuscript.

Field-specific reporting

Please select the one below that is the best fit for your research. If you are not sure, read the appropriate sections before making your selection.

☒ Life sciences ☐ Behavioural & social sciences ☐ Ecological, evolutionary & environmental sciences

For a reference copy of the document with all sections, see nature.com/documents/nr-reporting-summary-flat.pdf

Life sciences study design

All studies must disclose on these points even when the disclosure is negative.

Sample size	Sample size has been calculated for the main PRECISESADS project as followed (see Study Protocol below):<br>The study of distribution of expression value between 2 groups of patients allowed to generate hypotheses for the following parameters :<br>Type I error probability = 0.05<br>Type II error probability = 0.20 (i.e. Power 1— = 0.80)<br>Two-tailed tests<br>Allocation ratio = 9 (i.e. the cluster accounts for 10% of the total population)<br>Effect sizes: For t-tests on expression data: Cohen's d = 0.2 (i.e. difference of the means divided by standard deviation = 20%)<br>With these parameters, sample size estimations give: n1= 218 and n2 = 1966. Total N = 2184.<br>Where n1 is the sample size of the cluster, n2 the sample size of the remaining population, and N = n1 + n2 the total sample size.<br>On average, a total sample size of approximately 2000 patients (400 per disease) will allow identifying clusters of minimum 200 patients.<br>Our study population comprised 249 SSc patients and 365 Healthy Volunteers enrolled in the main PRECISESADS cross-sectional study
Data exclusions	Following complete quality control and diagnosis validation (in accordance with the 2013 ACR-EULAR classification criteria of the disease), 74 patients were removed (No RNAseq data or did not pass the RNAseq quality control criteria)
Replication	Due to blood sampling for multiple purposes, and volume/cell number limitations, data were not replicated in the independent human and mouse studies. Results were obtained from 12 individual animals in each experimental group of the mouse HOCl study. For lung and skin histology analyses in the mouse HOCl model, data were obtained from three independent sections in each tissue. Data on mAb characterization and binding characteristics were obtained from individual experiments. Wherever applicable, all experiments were carried out with relevant negative and positive controls or groups.
Randomization	Randomization in the human PRECISESADS cohort was not relevant since this is an observational study.<br>For the mouse HOCl study, the randomization strategy complied with the ARRIVE guidelines (https://arriveguidelines.org/arrive-guidelines/randomisation) and was based on the blocking method, taking into account only the cage location as a nuisance variable.
Blinding	Blinding was not feasible since this is an observational study.

Reporting for specific materials, systems and methods

We require information from authors about some types of materials, experimental systems and methods used in many studies. Here, indicate whether each material, system or method listed is relevant to your study. If you are not sure if a list item applies to your research, read the appropriate section before selecting a response.

Materials & experimental systems

n/a	Involved in the study
	☒ Antibodies
	☒ Eukaryotic cell lines
☒	Palaeontology and archaeology
	☒ Animals and other organisms
	☒ Clinical data
☒	Dual use research of concern
☒	Plants

Methods

n/a	Involved in the study
☒	ChIP-seq
	☒ Flow cytometry
☒	MRI-based neuroimaging

Antibodies

Antibodies used

- Polyclonal anti-Human IgG (Fab specific) peroxidase antibody, produced in goat (Sigma #A0293)
- Monoclonal anti-human c-Myc, produced in mouse, clone 9E10 (Santa Cruz #sc-40)
- Polyclonal anti-human Gal-3 IgG produced in rabbit (Abcam #ab53082)
- Polyclonal anti-human Gal-1 IgG produced in rabbit ((Abcam #ab25138)
- Polyclonal anti-human Gal-7 IgG produced in rabbit (Abcam #ab108623)
- Polyclonal anti-human Gal-2 IgG produced in goat (R&Dsystems #AF1153)
- Monoclonal anti-human Gal-4 IgG produced in mouse, clone 198616 (R&Dsystems #MAB1227)
- Monoclonal anti-human Gal-8 IgG produced in mouse, clone 210608 (R&Dsystems #MAB1305)
- Monoclonal anti-human Gal-10 IgG produced in mouse, clone 561603 (R&Dsystems #MAB5447)
- Monoclonal anti-human Gal-14 IgG produced in mouse , clone 511124 (R&Dsystems #MAB5744)
- Polyclonal anti-rabbit IgG, HRP-linked antibody (Cell Signaling #7074)
- Polyclonal anti-asialofetuin IgG, produced in sheep (Abcam #ab35184)
- Monoclonal anti-mouse F4/80 IgG, produced in rabbit, clone SP115 (Abcam #ab111101)
- Polyclonal anti-human IgG Fc, produced in goat (Millipore #AP113)
- Trastuzumab reference mAb, IgG1, #EU/1/00/145/001, same as original commercial pharmaceutical product
- Monoclonal mouse anti-human IgG CH2 secondary antibody, clone R10Z8E9 (ThermoFisher #MA5-16929)
- Monoclonal mouse anti-human IgG antibody, clone G18-145 (BD biosciences #555784)
- Polyclonal anti-human IgG Fc_biotin, produced in goat (Southern Biotech #2014-08)
- Polyclonal anti-mouse IgG (H+L), produced in goat, Alexa Fluor-conjugated (ThermoFisher #A-21235)
- Polyclonal anti-Mouse IgG Secondary Antibody, produced in goat, Alexa Fluor Plus 488-conjugated, (ThermoFisher #A32723)

Flow cytometry, mouse:
- Monoclonal rat anti-CD16/CD32 (Mouse Fc Block), clone 2.4G2 (BD Biosciences #553142)
- Monoclonal rat anti-CD45-APC, clone 30-F11 (Biolegend #103111)
- Monoclonal rat anti-CD45-FITC clone 30-11 (Biolegend #103107)
- Monoclonal rat anti-CD11b-APC-Cy7, clone M1/70 (BD Biosciences #561039)
- Monoclonal rat anti-CD3-PC7, clone 17A2 (Biolegend #100219)
- Monoclonal rat anti-CD4-PE, clone H129.19 (BD Biosciences #553653)
- Monoclonal rat anti-CD8-Pacific Blue, clone 53-6.7 (BD Biosciences #558106)
- Monoclonal rat anti-CD19-BV510, clone 1D3 (BD Biosciences #562956)
- Monoclonal rat APC anti-CD335, clone 29A1.4 (Biolegend #137607)
- Rat isotype control IgG2b-APC, clone RTK4530 (Biolegend #400611)
- Rat isotype control IgG2b-FITC, clone RTK4530 (Biolegend #400634)
- Rat isotype control IgG2b-APC-Cy7, clone A95-1 (BD Biosciences #552773)
- Rat isotype control IgG2b-PC7, clone RTK4530 (Biolegend #400617)
- Rat isotype control IgG2a-PE, clone R35-95 (BD Biosciences #553930)
- Rat isotype control IgG2a-Pacific Blue, clone R35-95 (BD Biosciences #558109)
- Rat isotype control IgG2a-BV510, clone R35-95 (BD Biosciences #562952)
- Rat isotype control IgG2a-APC, clone RTK2758 (Biolegend #400511)

Flow cytometry, human: the following antibodies were used for immunophenotyping of SSc patients and HV of the PRECISESADS cohort (published in Barturen, G. et al., Integrative analysis reveals a molecular stratification of systemic autoimmune diseases. Arthritis Rheumatol. 73, 1073-1085 (2021); Soret, P. et al. A new molecular classification to drive precision treatment strategies in primary Sjögren's syndrome. Nat. Commun. 12, 3523 (2021); Le Lann, L. et al. Standardization procedure for flow cytometry data harmonization in prospective multicenter studies. Sci. Rep. 10, 11567 (2020); Jamin, C. et al. Multi-center harmonization of flow cytometers in the context of the European "PRECISESADS" project. Autoimmun. Rev. 15, 1038-1045 (2016).

Duraclone panel (Beckman) antibodies used comprised:
- CD15-PE, IgM clone 80H5 (Beckman #IM1954U)
- CD16-FITC, IgG1 clone 3G8 (Beckman #IM6604894)
- CD19-APC, IgG1 clone J3-119 (Beckman #IM2470)
- CD3-APC-A750, IgG1 clone UCHT1 (Beckman #A94680)

Validation

- Anti-Human IgG (Fab specific) Peroxidase antibody (Sigma, A0293, goat polyclonal) – validation of Fab selectivity by ELISA and human reactivity by IHC on FFPE human tonsil.
- Anti-human c-Myc monoclonal antibody produced in mouse (Santa Cruz, sc-40) - validation of myc selectivity by Western blot on whole cell lysates prepared from non-transfected cells and c-Myc fusion protein transfected cells and human reactivity by IHC on FFPE human tissues.
- Anti-human Gal-3 polyclonal IgG (Abcam, ab53082) - validation of Gal-3 selectivity by Western blot on cell extract and cell extract with blocking peptide and human reactivity by IHC on FFPE human lung.
- Anti-human Gal-1 polyclonal IgG produced in rabbit (Abcam, ab25138) - validation of Gal-1 selectivity by Western blot on cell

lysates and human reactivity by IHC on FFPE human placenta.
- Anti-human Gal-7 polyclonal IgG produced in rabbit (Abcam, ab108623) - validation of Gal-7 selectivity by Western blot on cell lysate and human reactivity by IHC on FFPE human tonsil.
- Anti-human Gal-2 polyclonal IgG (R&Dsystems, AF1153, goat polyclonal) – human Gal-2 selectivity, and less than 1% cross-reactivity with recombinant human Galectin-1, -4, -7, and -8 are observed in direct ELISA and Western blot
- Anti-human Gal-4 monoclonal IgG produced in mouse (R&Dsystems, MAB1227) - human Gal-4 selectivity in direct ELISA and Western blot on cell lysates. No cross-reactivity with recombinant human Galectin-1, -2, -3, -7, -8, and recombinant mouse Galectin-1, -3, -4, or -7 is observed in direct ELISA.
- Anti-human Gal-8 monoclonal IgG produced in mouse (R&Dsystems, MAB1305) - human Gal-8 selectivity and no cross-reactivity with recombinant human Galectin-1, -2, or -7 are observed in direct ELISA and Western blot.
- Anti-human Gal-10 monoclonal IgG produced in mouse (R&Dsystems, MAB5447) - human Gal-10 selectivity in direct ELISA and Western blot. No cross-reactivity with recombinant human Galectin-1, -2, -3, -4, -7, -8, or -9/Ecalectin is observed in direct ELISA.
- Anti-human Gal-14 monoclonal IgG produced in mouse (R&Dsystems, MAB5744) - human Gal-14 selectivity in direct ELISA and Western blot. No cross-reactivity with recombinant human Galectin-1, -2, -3, -4, -7, -8, -10, or recombinant mouse Galectin-9 is observed in direct ELISA.
- Anti-rabbit IgG, HRP-linked antibody (Cell Signaling, 7074, goat polyclonal) used for Elisa, Western blot or Immunohistochemistry application in more than 1 000 publications
- Anti-asialofetuin polyclonal IgG (Abcam, ab35184, sheep polyclonal) - recommended for ELISA applications, cow asialofetuin selectivity by ELISA checked with purified full length native protein.
- Monoclonal anti-mouse F4/80 IgG, produced in rabbit (Abcam #ab111101) - recommended for immunohistochemistry applications. Positive control: Mouse colon, liver and lung tissue; M1 and M2 macrophages from mice colon tissue. Used in more than 120 publications.
- Polyclonal anti-human IgG Fc, produced in goat (Millipore #AP113), key applications in ELISA, IP, WB for the detection of human IgG. Shows a single band when run in an IEP system at a minimum concentration of 10 mg/mL, using human whole serum as the antigen. Based on immunoelectrophoresis, the antibody reacts with the heavy chains on human IgG but not with the light chains on most human immunoglobulins.
- Trastuzumab reference mAb, IgG1, #EU/1/00/145/001, same as original commercial pharmaceutical product
- Monoclonal mouse anti-human IgG CH2 secondary antibody, (ThermoFisher #MA5-16929), validated for ELISA and Western blot applications. Western blot (non-reducing) was performed using #MA5-16929 and a 150 kDa band corresponding to human IgG was observed in IM-9, ARH-77 and IM-9, ARH-77 conditioned medium8203 (CM) but not in Raji, Jurkat, Molt-4 and Jurkat CM which are known to have low expression.
- Monoclonal mouse anti-human IgG antibody (BD biosciences #555784), recommended for flow cytometry and ELISA applications. Specifically binds to the heavy chain of human immunoglobulin G subclasses: IgG1, IgG2, IgG3 and IgG4. Does not react with the heavy chains of other human immunoglobulin isotypes.
- Polyclonal anti-human IgG Fc_biotin, produced in goat (Southern Biotech #2014-08), minimal reactivity to rabbit, mouse, rat, bovine, horse, hamster, goat, sheep, chicken, and guinea pig serum proteins, validated for use in ELISA and flow cytometry assays.
- Polyclonal anti-mouse IgG (H+L), produced in goat, Alexa Fluor-conjugated (ThermoFisher #A-21235). Described for IHC and flow cytometry applications. Affinity purified and cross-adsorbed against human IgG and human serum prior to conjugation. Cross-adsorption or pre-adsorption is a purification step to increase specificity of the antibody resulting in higher sensitivity and less background staining.
- Polyclonal anti-Mouse IgG Secondary Antibody, produced in goat, Alexa Fluor Plus 488-conjugated, (ThermoFisher #A32723), described in western blot, immunofluorescence and immunohistochemistry applications. To minimize cross-reactivity, the goat anti-mouse IgG whole antibodies have been pre cross-adsorbed against bovine IgG, goat IgG, rabbit IgG, rat IgG, human IgG, and human serum. Cross-adsorption or pre-adsorption is a purification step to increase specificity of the antibody resulting in less background staining and cross-reactivity.
- Flow cytometry, mouse:
All antibodies were purchased from commercial sources (Biolegend or BD Biosciences) and were validated by manufacturer for the application: flow cytometry and the species: mouse.
Statement from BioLegend: Specificity testing of 1-3 target cell types with either single- or multi-color analysis (including positive and negative cell types). Once specificity is confirmed, each new lot must perform with similar intensity to the in-date reference lot. Brightness (MFI) is evaluated from both positive and negative populations. Each lot product is validated by QC testing with a series of titration dilutions.
Statement from BD Biosciences: The specificity is confirmed using multiple methodologies that may include a combination of flow cytometry, immunofluorescence, immunohistochemistry or western blot to test staining on a combination of primary cells, cell lines or transfectant models. All flow cytometry reagents are titrated on the relevant positive or negative cells. To save time and cell samples for researchers, test size reagents are bottled at an optimal concentration with the best signal-to-noise ratio on relevant models during the product development. To ensure consistent performance from lot-to-lot, each reagent is bottled to match the previous lot MFI.
- Flow cytometry, human:
The Duraclone panels include optimized antibody combinations for the identification and characterization of cells of the human immune system. Dry, unitized antibody panels, powered by DURA Innovations technology (https://www.beckman.com/resources/technologies/dura-innovations), include backbone markers for cell identification, opens slots for drop-in markers, and save time on designing immunophenotyping assays (https://www.beckman.com/reagents/coulter-flow-cytometry/antibodies-and-kits/duraclonepanels). Testing was performed on normal whole blood and/or cultured cell lines to confirm reactivity and specificity of each antibody.
Relevant citations:
Hedley BD, Keeney M, Popma J, Chin-Yee I. Novel lymphocyte screening tube using dried monoclonal antibody reagents. Cytometry B Clin Cytom. 2015 Nov-Dec;88(6):361-70. doi: 10.1002/cyto.b.21251. Epub 2015 Jul 17. PMID: 25944189.
Bouriche L, Bernot D, Nivaggioni V, Arnoux I, Loosveld M. Detection of Minimal Residual Disease in B Cell Acute Lymphoblastic Leukemia Using an Eight-Color Tube with Dried Antibody Reagents. Cytometry B Clin Cytom. 2019 Mar;96(2):158-163. doi: 10.1002/cyto.b.21766. Epub 2019 Jan 30. PMID: 30698327.

Eukaryotic cell lines

Policy information about cell lines and Sex and Gender in Research

Cell line source(s)	D11 and E07 mAbs were produced in CHO-K1 cells originally received from ATCC.
Authentication	Certified from provider. Karyotyping and a full genome sequencing was performed when the cells were sourced. Cell banks to use for production were ongoing regular checks for cell growth, viability, productivity, and occasional microscopy (morphology).
Mycoplasma contamination	CHO -K1 cells were not tested for mycoplasma for the purpose of mAb production.
Commonly misidentified lines (See ICLAC register)	None.

Animals and other research organisms

Policy information about studies involving animals; ARRIVE guidelines recommended for reporting animal research, and Sex and Gender in Research

Laboratory animals	6-week old Balb/c AnNRj mice.
Wild animals	The study did not involve wild animals.
Reporting on sex	Only female mice were used, as described previously (Kavian N. et al. Arthritis Rheum. 2010 (11):3477-87. doi: 10.1002/art.27626).
Field-collected samples	The study did not involve samples collected from the field.
Ethics oversight	All mice were maintained in a specific pathogen-free facility at the Pasteur Institute of Lille, fed with a standard diet and given free access to water at a temperature of 22 ± 2°C and a 35-70% humidity atmosphere, with 12 h light /12 h dark cycles. Animal experiments were performed according to the european guidelines N° 68/609/CEE (approval number: #19603-2020061914271271 v6). The mouse study was approved by the local ethics committee: Comité d'éthique en expérimentation animale (CEEA 75), Nord Pas-de-Calais, France.

Note that full information on the approval of the study protocol must also be provided in the manuscript.

Clinical data

Policy information about clinical studies

All manuscripts should comply with the ICMJE guidelines for publication of clinical research and a completed CONSORT checklist must be included with all submissions.

Clinical trial registration	The cross-sectional study is registered in ClinicalTrials.com with number NCT02890121.
Study protocol	Protocol Code: PRECISESADS GA-115565 CS<br><br>Molecular Reclassification to Find Clinically Useful Biomarkers for Systemic Autoimmune Diseases<br><br>The information in this document is confidential and it is property of the PRECISESADS Consortium, operating under the INNOVATIVE MEDICINES INITIATIVE JOINT UNDERTAKING (IMI-JU). Therefore, the information should not be disclosed, published, made public or otherwise transferred to a third party, in any form without written permission from the PRECISESADS Consortium. However, this document may be disclosed to researchers and potential researchers, health authorities and relevant national ethics committees under the condition that they respect the confidential nature of this document.<br><br>Objectives<br>The main objective of the PRECISESADS project is to reclassify the individuals affected by systemic autoimmune diseases (SAD) into molecular clusters instead of clinical entities through the determination of molecular profiles using several "-omics" techniques.<br><br>Design<br>This is a European multi center, non randomized, cross sectional clinical study aiming at collecting clinical and biological data on patients and healthy controls with SAD<br><br>Schedule of Events<br>Clinical assessments for the different populations of the study are detailed below:<br>n=400 patients with systemic lupus erythematosus<br>n=400 patients with rheumatoid arthritis<br>n=400 patients with systemic sclerosis<br>n=400 patients with Sjogren's syndrome<br>n=400 patients with primary antiphospholipid syndrome / mixed connective tissue disease<br>n=666 healthy controls (matched)<br>Informed consent for all<br>Inclusion/exclusion criteria for all

Demography for all
Clinical diagnosis for all
Clinical history for all
Disease activity for all
Current treatment for all
Clinical data (lab & Imaging data when available) for all
Clinical examination for all
Blood sample for all
Urine sample for all
Optional separated cell substudy blood sampling (depending on specific informed consent)

Sampling procedures
For all individuals
- Fresh whole blood for flow cytometry analyses (2 ml);
- Blood for processing to obtain plasma and DNA (20 ml);
- Blood for RNA (5 ml);
- Blood for serum (17 ml);
- Blood for lupus anticoagulant (2.7 ml)
- Urine (100 ml).
- Optional sampling for "separated cell populations" in 288 individuals (240 patients, 48 per disease and 48 controls) will be studied in more detail by allowing cell separation: an extra 80-100 ml sampling of fresh blood

Further detailed information is available in the Nature Communications summary report of Soret et al., A new molecular classification to drive precision treatment strategies in primary Sj6gren's syndrome. Nat. Commun. 2021 Jun 10;12(1):3523. available at https://static-content.springer.com/esm/art%3A10.1038%2Fs41467-021-23472-7/Media0bjects/41467_2021_23472_M0ESM18_ESM.pdf

Data collection

Recruitment from December 2014 to October 2017

19 recruiting clinical sites
1-Centro Hospitalar do Porto, Largo Prof. Abel Salazar 4099-001 PORTO (Portugal)
2-Fondazione IRCCS Ca Granda Ospedale Maggiore Policlinico via Francesco Sforza n.28 20122 Milano (Italy)
3-Hospital Clinic I Provicia- Institut d'Investigacions Biomediques August Pi i Sunyer Calle Villarroel 170 08036 Barcelona (Spain)
4-Hospital Universitario San Cecilio Servicio Andaluz de Salud Avda. del Dr. Oloriz n°16 18012 Granada (Spain)
5-Hospital Universitario Reina Sofia Andaluz de Salud Avda. Menendez Pidal, s/n 14004 Cordoba (Spain)
6-Hospital Universitario Marques de Valdecilla, Servicio Cantabro de Salud Avd. Cardenal Herrera Oria s/n, 39011 Santander, (Spain)
7-UNIMI, Istituto Ortopedico Getano Pini, Piazza A. Ferrari 1, 20122 Milano (Italy)
8-University of Szeged, H-6720 Szeged, Dugonics square 13 (Hungary)
9-Medical University of Vienna Spitalgasse 23, 1090 Wien (Austria)
10-Hospital Regional de Malaga Servicio Andaluz de Salud Avda. Carlos Haya s/n 29010 Malaga (Spain)
11-Hospitaux Universitaires de Geneve Rue Gabrielle-Perret-Gentil 4, 1205 Geneve (Switzerland)
12-Centre Hospitalier Universitaire de Brest Hospital de la Cavale Blanche Boulevard Tanguy Prigent CP : 29609 Brest CEDEX, (France)
13-UZ Leuven - KU Leuven, Department of Rheumatology Herestraat 49, 3000 Leuven (Belgium)
14-Deutsches Rheuma-Forschungszentrum Berlin Charitestra8e 1, 10117 Berlin (Germany)
15-Medizinische Hochschule Hannover Carl-Neuberg-Str. 1 30625 Hannover (Germany)
16-Hospital Virgen de las Nieves Granada Avenida de las Fuerzas Armadas, 2, 18014, Granada (Spain)
17-University catholique de Louvain —Cliniques Universitaires Saint-Luc Avenue Hippocrate 10, 1200 Brussels (Belgium)
18-University of Cologne, Dept. of Dermatology, Kerpener Str. 62, 50937, Cologne (Germany)
19-Andalusian Public Health System Biobank, Granada (Spain).

Laboratory sample processing by: The Andalusian Biobank.
FPS, BAYER, DRFZ, IDIBELL, CSIC, KU LEUVEN, ALTHIA, KI, UGR, UNIMI, UBO
January 2015-May 2015
Data analysis of first sets of processed materials, and optimization of protocols according to preliminary results
Coordinating investigator and sample analysis sites : FPS, IDIBELL, DRFZ, UBO, KI, CSIC, ALTHIA, UGR, UNIMI, BAYER, QBIO
January 2015-December 2017
Analysis of samples and data by research sites (bioinformatical or genetic analysis, statistical) FPS, IRIS, QBIO, IDIBELL, UBO, UGR, CSIC, ALTHIA, DRFZ, BAYER, KI, UKLEUVEN, UNIMI
January 2015-January 2019
Data analysis ongoing throughout the project and dissemination of the results FPS, UCB, CSIC, IDIBELL, UBO, DRFZ, CIBIO, BAYER, IRIS, SARDS, KI, UNIMI
Further detailed information is available in the Nature Communications summary report of Soret et al., A new molecular classification to drive precision treatment strategies in primary Sj6gren's syndrome. Nat. Commun. 2021 Jun 10;12(1):3523. available at https://static-content.springer.com/esm/art%3A10.1038%2Fs41467-/Media0bjects/41467_2021_23472_M0ESM18_ESM.pdf

Outcomes

Data produced from individuals with SAD and controls: genetic, epigenomic, transcriptomic, flow cytometry data, plasma and urine metabolomics, serum cytokine analyses, and serology (autoantibodies).
- Total blood for flow cytometry for measurement of cell proportions : done on site at each clinical site
with 8 or 10-color flow cytometers (all sites except MUW, SCS, USZ and SAS Malaga which will transfer the blood sample to the closest equipped site).
- Blood for processing to obtain DNA and plasma: tubes processed to obtain an aliquot of plasma; cell pellet frozen and sent to the Andalusian biobank for the extraction of DNA.
- Preserved blood to obtain total RNA: tubes frozen and sent to the Andalusian biobank
- Blood for processing to obtain serum: tube centrifuged, serum obtained and transferred to smaller tubes, aliquoted and frozen and sent to the Andalusian Biobank for storage.
- Blood with citrate for measuring lupus anticoagulant: tubes centrifuged, plasma obtained and transferred tov smaller tubes, frozen

and sent to the Andalusian Biobank for storage.
- Urine: processed according to instructions, aliquoted and sent to the Andalusian biobank.
Further detailed information is available in the Nature Communications summary report of Soret et al., A new molecular classification to drive precision treatment strategies in primary Sj6gren's syndrome. Nat. Commun. 2021 Jun 10;12(1):3523. available at https://static-content.springer.com/esm/art%3A10.1038%2Fs41467-021-23472-7/MediaObjects/41467_2021_23472_MOESM18_ESM.pdf

Flow Cytometry

Plots

Confirm that:

☒ The axis labels state the marker and fluorochrome used (e.g. CD4-FITC).

☒ The axis scales are clearly visible. Include numbers along axes only for bottom left plot of group (a 'group' is an analysis of identical markers).

☒ All plots are contour plots with outliers or pseudocolor plots.

☒ A numerical value for number of cells or percentage (with statistics) is provided.

Methodology

Sample preparation

- Human
Among the critical points reducing the reproducibility during flow cytometry studies are the instability of the fluorochromes conjugated to the antibodies (Abs) mainly of the tandem fluorochromes, and the pipetting errors of the reagents that may lead to changes in staining levels. To bypass these problems, we used Duraclone tubes (Beckman Coulter) specifically designed and optimized for the PRECISESADS study. These tubes correspond to ready-to-use unitized, dry format Ab cocktails. They eliminate errors due to manual Ab preparations, they improve the stability compared to liquid reagents, avoiding tandem breakdown, and are room temperature stable, thus excluding the need to manage varying expiration date and revalidations among single color liquid Abs.
Detailed standard operating procedures for sample staining with the Duraclone tubes have been already published in Table 5 of the following manuscript: (Jamin C. et al., Multi-center harmonization of flow cytometers in the context of the European "PRECISESADS" project. Autoimmun Rev. 2016 Nov;15(11):1038-1045. doi:10.1016/j.autrev.2016.07.034.)
Staining :
1. Prepare the IOTest 3 lysing 1 x/fixative solution (Beckman Coulter):
a. Dilute the IOTest 3 Lysing Solution 10 x in distilled water.
b. Add the IOTest 3 Fixative Solution according to the manufacturer's instructions.
c. Mix well.
2. Drop by reverse pipetting the required volume of blood samples (either 50 µL or 100 µL) as indicated in the Standard Operating Procedure into the corresponding Duraclone tubes:
a. Mix for 10 s to rehydrate the antibodies.
b. Incubate the tubes for 20 min at room temperature in the dark.
3. Add 2 mL of the IOTest 3 lysing 1 x/fixative solution at room temperature.
a. Mix for 2 s.
b. Incubate the tubes for 20 min at room temperature in the dark.
4. Add the FlowCount fluorospheres (Beckman Coulter).
a. Mix the vial for 5 s and shake it 5 times by inversion.
b. Add by reverse pipetting 50 µL or 100 µL of fluorospheres (same volume as blood with the same pipet).
c. Mix for 2 s.
5. Tubes are ready for acquisition
a. keep at 4°C in the dark until acquisition
b. Acquire the cells within 2 h after preparation

- Mouse
The BAL fluid from the three lavages was pooled and processed for FACS analysis. The BAL fluid was centrifuged at 400 x g for 7 min at 4°C. The supernatant was harvested and stored at -80°C. Cell pellets were incubated for 2 min in Red Blood Cell Lysing Buffer (Hybri-Max, Sigma-Aldrich) and washed in PBS-SVF 2%-EDTA 1 mM. Cells were resuspended in 130 µL of PBS-SVF 2%-EDTA 1 mM containing 10 µg/mL Fc Block (clone 2.4G2; cat. 553142, BD Biosciences) and incubated at 4°C for 5 min. For the numeration of viable CD45+ cells in BAL fluid, cell suspensions (50 µL) were incubated with anti-CD45-APC antibody or the corresponding isotype control for 20 min at 4°C, protected from light, and with propidium iodide for 5 min. Flow-Count Fluorospheres (Beckman Coulter) were added before data acquisition.

Instrument

-Human
Multi-parameter flow cytometry analyses were conducted in 11 different centers from the PRECISESADS consortium. Therefore, the integration of all data in common bioinformatical and biostatistical investigations has required a fine mirroring of all instruments (Le Lann Let al., Standardization procedure for flow cytometry data harmonization in prospective multicenter studies. Sci Rep. 2020 Jul 14;10(1):11567).
The calibration procedure elaborated to achieve this prerequisite and the antibody panels used have been described previously (Jamin C et al., Multi-center harmonization of flow cytometers in the context of the European "PRECISESADS" project. Autoimmun Rev. 2016 Nov;15(11):1038-1045). In brief, in the 11 sites responsible for the flow cytometry acquisition, a Navios flow cytometer (Beckman Coulter) was used in 3 centers, a Gallios (Beckman Coulter) in 1 center, a FACS Canto II (BD Biosciences) in 4 centers and a FACS Aria III, a FACS Verse and a LSR Fortessa (BD Biosciences) in 1 center each. All instruments are equipped with 3 lasers emitting at 405/407, 488, and 633/635 nm, and with optical filter configuration permitting the detection of FITC, PE, APC and APC-A750 fluorochromes. VersaComp Ab capture bead kit (Beckman Coulter) is

used for the photomultiplier tube adjustments and the determination of the target MFI values applied for the multicenter harmonization procedure. Eight-peak rainbow bead calibration particles (Spherotech) are used over the 5-year duration of the study for the daily checks as monocenter verification of the instrument stability. The same lots of VersaComp capture beads (#4,131,003K) and of 8-peak rainbow beads (#AF01) were ordered in all centers.

- Mouse
Data were acquired on a 4-laser cytometer (CytoFLEX, Beckman Coulter)

Software

-Human
The strategy developed to avoid any redundancy in the different cell subsets and to increase the accuracy of the phenotypes has been automated by AltraBio (Lyon, France), under subcontracting. The automation of gating has been built using a supervised Machine learning-based approach using training datasets gated manually. This automation required a 2-step workflow. The first step was customized for each flow cytometer due to potential differences in signal for the Forward Scatter and Side Scatter (FS/SS) measures across the different instruments used in the study. The second non-instrument specific step was for gating all remaining populations of interest. In order to validate the Machine learning-based algorithms (name automatons), intermediate evaluations were carried out. The results generated by the automatons were compared with manual gating analysis performed by the same operator with the Kaluza software on 300 patients distributed throughout the centers. The comparison of the results showed a very good correlation of the data of frequencies, absolute values and the MFI (coefficient of correlation 0.9996), making it possible to validate the efficiency of the automatons. This strategy has been published previously: Le Lann Let al., Standardization procedure for flow cytometry data harmonization in prospective multicenter studies. Sci Rep. 2020 Jul 14;10(1):11567.

- Mouse
After acquisition on a 4-laser cytometer (CytoFLEX, Beckman Coulter), data were analyzed with dedicated software (Kaluza v2.1, Beckman Coulter).

Cell population abundance

No sort in this paper. For the human study, the distribution of the different cell subsets is provided in Supplementary Figure 3 (Le Lann L, Jouve PE, Alarcon-Riquelme M, Jamin C, Pers JO; PRECISESADS Flow Cytometry Study Group; PRECISESADS Clinical Consortium.Standardization procedure for flow cytometry data harmonization in prospective multicenter studies. Sci Rep. 2020 Jul 14;10(1):11567. doi: 10.1038/s41598-020-68468-3)

Gating strategy

- Human
The gating strategy has been published in details in: Le Lann L. et al., Standardization procedure for flow cytometry data harmonization in prospective multicenter studies. Sci Rep. 2020 Jul 14;10(1):11567.
After exclusion of debris, dead cells and doublets, neutrophils (CD15hiCD16hi) were gated from CD15+ polymorphonuclear cells. Lymphocytes were identified as B cells (CD19+CD3-) and T cells (CD19-CD3+).
- Mouse: The gating strategy is shown in Supplementary Figure 13 of the current manuscript.
CD45 was used to distinguish leukocytes, single cells were selected based on FSC-A vs FSC-H, dead cells were then removed according to 7-AAD staining. Among the cells FSClow with low autofluorescence, B cells, NK cells and T cells were gated based on expression of CD19, CD335 and CD3, respectively. Among T cells, CD4 vs CD8 allowed to distinguish CD4+ T cells and CD8+ T cells. Among CD19- CD3- CD335- cells: autofluorescence and CD11b discriminated alveolar macrophages (autofluorescencehigh CD11b-/low) and other myeloid cells: monocytes, dendritic cells, neutrophils and eosinophils (autofluorescencelow/int CD11b+/high).

☒ Tick this box to confirm that a figure exemplifying the gating strategy is provided in the Supplementary Information.
